# Decoding plant defense signaling using the *defenseless* mutant

**DOI:** 10.1111/nph.70939

**Published:** 2026-01-23

**Authors:** Bikash Baral, Mikael Brosché

**Affiliations:** ^1^ Department of Organismal and Evolutionary Biology, Faculty of Biological and Environmental Sciences, Viikki Plant Science Centre University of Helsinki FI‐00014 Helsinki Finland

**Keywords:** defense mechanism, defense regulators, immunity, interactions, reactive oxygen species, RNA‐seq, signaling cascades

## Abstract

- Can plants live without defenses? Mutant analysis in *Arabidopsis thaliana* has identified numerous regulators of biotic, abiotic, and hormone‐based defenses, but the redundancy among separate defense pathways remains unexplored.
- We constructed an Arabidopsis mutant, *defenseless*, lacking six canonical defense pathways using *abi1‐1* (abscisic acid), *coi1* (jasmonic acid), *sid2* (salicylic acid), *ein2* (ethylene), *eds1* (biotic defense signaling), and *rbohD* (apoplastic reactive oxygen species production), enabling dissection of defense network resilience.
- In optimal growth conditions, *defenseless* exhibited no stress phenotypes, demonstrating that plant defenses are dispensable under favorable environments. Stress assays revealed paradoxical responses: some defenses remained functional in *defenseless*, while others were severely compromised. Notably, ozone‐triggered apoplastic ROS signaling was largely preserved, uncovering alternative and redundant defense mechanisms.
- Transcriptome profiling identified a core set of immune‐related genes consistently downregulated in *defenseless*, yet pathogen susceptibility was not elevated beyond known immunity‐deficient mutants, underscoring extensive redundancy and positioning *defenseless* as a platform to probe several layers of plant defenses.

## Introduction

Immunodeficiency renders organisms highly vulnerable to a wide range of pathogens, as demonstrated by the emergence of acquired immunodeficiency syndrome, first identified in humans in the early 1980s (Bosma *et al*., 1983). Development of mice that lack the adaptive immune system is a key resource to understand disease in animals and humans (Walsh *et al*., 2017). In plants, protection against invaders relies on complex signaling networks for pathogen recognition and defense activation (Dodds *et al*., 2024). Plants are constantly exposed to pathogenic threats, both above‐ and belowground (De Coninck *et al*., 2015). Moreover, plants are also exposed to abiotic stress from changes in the environment, including drought, temperature extremes, air pollutants, and excess light (Zhang *et al*., 2022).

Plants are effective in combating invading pathogens, and only a fraction of these invaders are successful in causing disease (Wei *et al*., 2015; Saur & Hückelhoven, 2021). Plant defenses are activated by both biotic and abiotic stressors and rely on several signaling pathways that allow the plant to adapt to different stressors (Zhang *et al*., 2022; Dodds *et al*., 2024). Several hormones, jasmonic acid (JA), salicylic acid (SA), abscisic acid (ABA), and ethylene (ET) – play central roles in orchestrating these responses (Xu *et al*., 2015; Overmyer *et al*., 2018; Sun & Zhang, 2022; Zhang *et al*., 2022; Dodds *et al*., 2024). JA and SA regulate defense against pathogens (Ma & Ma, 2016), while ABA primarily regulates responses to environmental stress (Zhang *et al*., 2022). Plants detect invaders through pattern‐recognition receptors, which recognize microbe‐associated molecular patterns (MAMPs) and damage‐associated molecular patterns, initiating pattern‐triggered immunity (Dodds *et al*., 2024). Pathogens combat plant defenses by using effector proteins, where in turn, plants can recognize the effector to activate effector‐triggered immunity (ETI; Remick *et al*., 2023), which provides a stronger response, sometimes including localized cell death known as the hypersensitive response (Dalio *et al*., 2021). In the signaling pathway following pathogen perception by surface receptors or intracellular nucleotide‐binding leucine‐rich repeat, the lipase‐like protein ENHANCED DISEASE SUSCEPTIBILITY1 (EDS1) has a key function to link pathogen perception to downstream responses (Dongus & Parker, 2021; Dodds *et al*., 2024). EDS1 together with interacting partners PHYTOALEXIN DEFICIENT 4 (PAD4) or SENESCENCE‐ASSOCIATED GENE 101 activate defense signaling, programmed cell death and calcium (Ca^2+^) channels. In defense toward abiotic stress, plants rely on various sensing mechanisms, for example changes in turgor pressure that activate Ca^2+^‐channels (Zhang *et al*., 2022). The sensing mechanisms take place throughout the cell and include cell wall, plasma membrane, chloroplast, and mitochondria (Zhang *et al*., 2022). After perception of abiotic or biotic stress, downstream signaling components include reactive oxygen species (ROS), Ca^2+^ and kinases (Waszczak *et al*., 2018; Luan & Wang, 2021; Sun & Zhang, 2022; Dodds *et al*., 2024). Outputs from signaling pathways include activation of transcription factors and transcriptional reprogramming tailored to combat the specific stress.

ROS include hydrogen peroxide (H_2_O_2_) and superoxide (O_2_^·−^), and ROS have several roles during plant defense; they can strengthen cell walls and be toxic to invading pathogens, and they can act as signaling molecules during defense and plant development (Waszczak *et al*., 2018; Bleau & Spoel, 2021; Mittler *et al*., 2022). There are several challenges in understanding how ROS act as signals: (1) ROS are generated at multiple cellular locations, including the apoplast, mitochondria, chloroplast, peroxisome, and cytosol. (2) ROS are produced through diverse mechanisms, such as metabolic processes and catalytic formation by RESPIRATORY BURST OXIDASE HOMOLOGUEs (RBOHs). (3) ROS are perceived through specific molecular mechanisms, for example, via cysteine modifications in proteins (Waszczak *et al*., 2018). One way to start addressing the role of ROS in signaling is to use treatments in which the ROS are formed at a specific cellular location, rather than a general treatment that raises ROS at several places. Another approach is to use mutants defective in specific subcellular ROS production or scavenging. For example, extensive work with the peroxisomal *cat2* (*catalase 2*) has established how peroxisomal H_2_O_2_ contributes to plant defenses and development (Yang *et al*., 2019; Baker *et al*., 2023). In defense signaling, and especially plant–pathogen interactions, ROS produced in the apoplast are considered a key signal, exemplified by extensive regulation of RBOH enzymes that produce superoxide in the apoplast (Castro *et al*., 2021). Both RBOHD and RBOHF contribute to ROS used for signaling in abiotic and biotic stress (Torres *et al*., 2002) but *RBOHD* transcription levels are higher than *RBOHF* transcription levels (Morales *et al*., 2016), and RBOHD appears to be more important for producing ROS used in cell‐to‐cell communication (Miller *et al*., 2009; Castro *et al*., 2021). A direct approach to study the signaling function of apoplastic ROS is to apply the air pollutant ozone (O_3_). Ozone enters the plant via stomata and, in the cell wall, breaks down into hydrogen peroxide and superoxide; thus, ozone provides a tool to directly activate apoplastic ROS signaling without the need for any other manipulation of the plant (Xu *et al*., 2015; Waszczak *et al*., 2018).

As the global threat of phytopathogens and abiotic stress intensifies due to climate change, food security is at risk (Zandalinas & Mittler, 2022). A challenge to understand plant defenses is the redundancy and multiple different defense signaling pathways. For example, using higher‐order Arabidopsis mutants where several hormone and defense signals are removed uncovered that if one hormone signal is removed, compensatory mechanisms are present that still provide defenses (Hillmer *et al*., 2017). Inspired by animal models, where the entire immune system can be compromised (Walsh *et al*., 2017), we tested whether the same could be done in Arabidopsis by combining several mutations in key defense and hormone‐related genes. A plant without defenses would then act as a platform to study how plant defenses toward abiotic and biotic stress are organized.

**Fig. 1:**
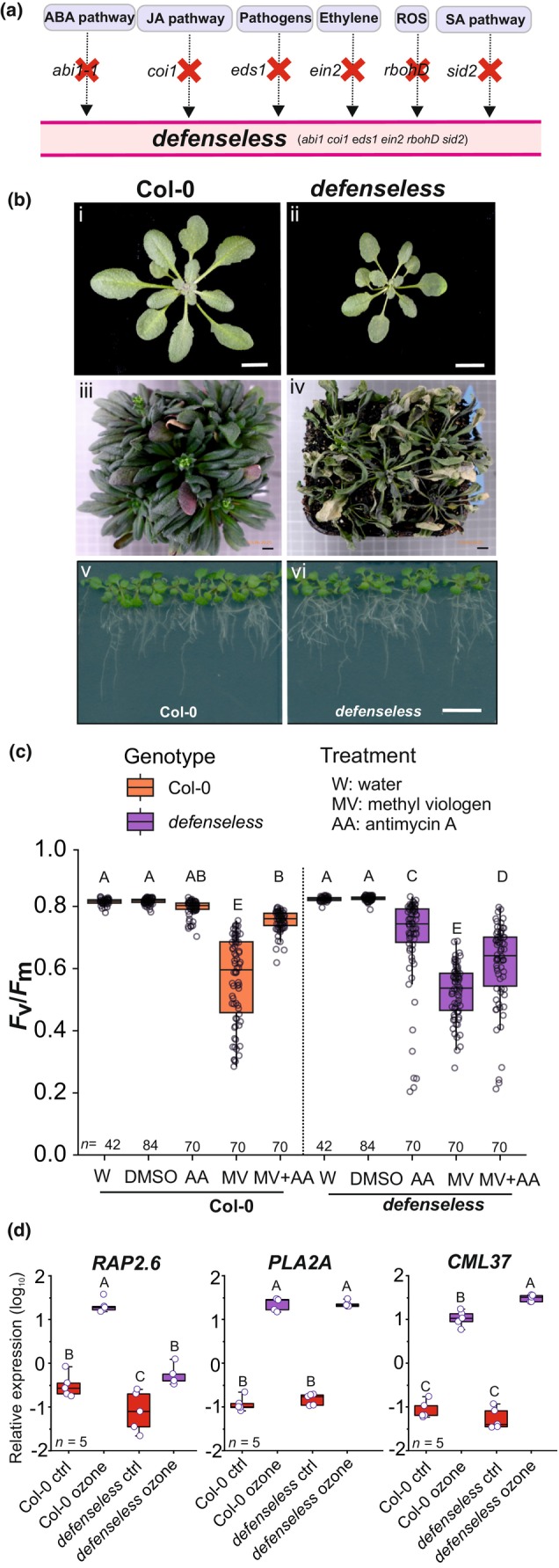
Overview of pathway disruptions in *defenseless* and its growth and stress phenotypes. (a) Schematic overview of the signaling pathways disrupted in the *defenseless* mutant. (b) Phenotypic comparison between Columbia‐0 (Col‐0) and *defenseless*: (i, ii) images showing 3 wk old Col‐0 and *defenseless* under standard growth conditions; (iii, iv) when grown in growth chambers for seed production, the *defenseless* mutant occasionally shows complete collapse and dying, (v, vi) root growth of Col‐0 and *defenseless* on ½ MS‐medium. (c) Leaf disk assay of Col‐0 and *defenseless* under different treatments with methyl viologen (MV), which creates reactive oxygen species (ROS) in chloroplast, and antimycin A (AA) that inhibits mitochondrial complex III. Data from seven biological repeats are presented as box‐and‐whisker plots (*n* = the number of leaf disks). The boxes represent the 25^th^ to the 75^th^ percentiles, with the horizontal line plotted at the median values. The individual data points are shown as circles, and the whiskers represent the minimum and maximum values. Statistical significance was determined using two‐way ANOVA, followed by Tukey's test (*P* < 0.05). Samples with different letters are significantly different. (d) Reverse transcription quantitative polymerase chain reaction (RT‐qPCR) was used to assess the relative expression of marker genes under control and ozone (see Supporting Information Fig. S1b for additional genes). Data represent five biological replicates, and statistical significance was determined using two‐way ANOVA, followed by Tukey's test (*P* < 0.05). Samples with different letters are significantly different.

Here, we describe the *defenseless* mutant (*abi1‐1 coi1 eds1 ein2 rbohD sid2*; Fig. 1a), lacking signaling or biosynthesis of hormones (ABA, ET, JA, and SA), pathogen signaling (EDS1), and apoplastic ROS production (RBOHD). With this set of mutants, we expect to remove defense signaling, but not necessarily all plant defenses, as defenses also rely on various other mechanisms, including antioxidants, secondary metabolites, and barriers, including the cell wall and cuticle (Göhre & Robatzek, 2008; Waszczak *et al*., 2018; Zaynab *et al*., 2018). We use *defenseless* in multiple assays related to biotic and abiotic stress, intra‐organellar signaling, and transcriptomics to define the contribution of these key regulators toward plant defenses. While *defenseless* was compromised in plant defenses, it also retained a largely intact transcriptome response, indicating that the plant defense signaling network is redundant, and it is difficult to eliminate plant defense signaling.

## Materials and Methods

### Plant lines and growth conditions

Seeds of *Arabidopsis thaliana* Columbia‐0 (Col‐0) and mutants were obtained from Nottingham Arabidopsis Stock Centre or were gifts from Prof. Jane Parker (*eds1‐2* in Col‐0), Dr Miguel Torres (*rbohD*), and Prof. Julian Schroeder (*abi1‐1* in Col‐0). We initially made the mutants *coi1 ein2 sid2* (Xu *et al*., 2015) and *coi1 eds1 ein2 sid2* (Overmyer *et al*., 2018). As *coi1‐16* is partially male sterile, higher‐order mutants containing *coi1‐16* were used as pollen acceptors in crosses to *abi1‐1* and *rbohD* to generate the sextuple *abi1‐1 coi1‐16 eds1‐2 ein2‐1 rbohD sid2‐1*, which we will refer to as the *defenseless* mutant. All the single mutants have been extensively studied for their function in plant defense and hormone signaling (Supporting Information Table S1). Identification of higher‐order mutants was based on initial screenings for hormone phenotypes: *ein2* was identified via the triple response, *coi1‐16* was identified via MeJA root growth assay and *abi1‐1* was identified via ABA root growth assay. Subsequently, the presence of all mutations were identified with PCR‐based markers (Table S1).

Seeds were sown on 1 : 1 peat/vermiculite, stratified for 3 d, and then grown at 22°C/19°C for a week. Seedlings were then transplanted into a new 1 : 1 peat/vermiculite mixture. All plants were grown in a controlled chamber (Weiss Bio1300; Weiss Gallenkamp) or growth rooms, at 22°C/19°C, in relative humidity of 60/90%, under a 12 h : 12 h, light : dark cycle, at 220–250 μmol m^−2^ s^−1^ light intensity. Experiment details for stress experiments and transcriptome analysis are provided in the Methods S1.

### Data analysis

All data analyses were performed using the R software (v.4.4.0; R Core Team, 2024; https://www.R‐project.org) or Graphpad Prism 10.4.2. To account for inter‐replicate variability, linear mixed‐effects models were implemented via the ‘lmer’ function from the ‘lme4’ package (Bates *et al*., 2015), incorporating biological replicates as random effects. Post hoc pairwise comparisons were conducted using Tukey's honest significant difference (HSD) method as implemented in the ‘emmeans’ package (Lenth & Piaskowski, 2017), to adjust for multiple tests and accurately identify significant group‐wise differences. A *P*‐value of < 0.05, < 0.01, and < 0.001 was considered to denote significant, moderately significant, and highly significant differences, respectively.

## Results

### Construction of the *defenseless* mutant

Plant defenses to abiotic and biotic stresses are dependent on plant hormones ABA, ET, JA, SA, and on small signaling molecules like ROS. In plant–pathogen signaling, after recognition of the pathogen, EDS1 is an essential signaling intermediate required for activation of defenses (Locci *et al*., 2023). We aimed to generate a plant with removed signaling (or biosynthesis) from hormones, ROS, and EDS1. The selected mutants should have strong impairment, but at the same time, the final plant with all mutations should also be viable and produce seeds. We selected the following mutants (Fig. 1a): *abi1‐1* that has strongly impaired ABA signaling (Merilo *et al*., 2013); *coi1‐16*, a conditional allele for the COI1 JA receptor (Ellis & Turner, 2002) – as complete knockouts of COI1 are infertile, the use of *coi1‐16* allows seeds to be produced; *eds1‐2* that lacks EDS1 (Bartsch *et al*., 2006); *ein2‐1* with impaired ET signaling (Alonso *et al*., 1999); *rbohD* that lacks the apoplastic ROS producer RBOHD (Torres *et al*., 2002; Miller *et al*., 2009); and *sid2‐1* impaired in the main Arabidopsis SA biosynthesis pathway (Wildermuth *et al*., 2001). We used the JA receptor *coi1* rather than a JA biosynthesis mutant (e.g. *aos*, *allene oxide synthase*), as biosynthesis mutants also lack intermediates that can act as signals (Wasternack & Feussner, 2018). The focus of this work was to characterize *defenseless* (*abi1‐1 coi1 eds1 ein2 rbohD sid2*), but where appropriate single mutants (especially *abi1‐1*), and other mutants defective in production of defense molecules were used for comparison.

When grown in clean growth rooms or chambers, the *defenseless* mutant has no visible signs of stress (Fig. 1b). The leaves of *defenseless* are narrower and smaller than Col‐0 and are similar to the *abi1‐1* single mutant. We quantified fresh weight, which confirmed the smaller size of *defenseless* (Fig. S1a). The smaller size of *defenseless* and *abi1‐1* probably reflects the role of hormones as regulators of growth, particularly ABA (Cutler *et al*., 2010). Although *defenseless* can complete the entire life cycle to produce seeds, it is a poor seed producer, like the single *coi1‐16*. While *defenseless* showed normal growth in clean growth conditions, we observed occasional collapse and dying of the plants (Fig. 1b) and moving of plants from the growth chamber to the glasshouse often led to infection from (yet unidentified) opportunistic pathogens (Fig. S1e). Growing plants in sterile *in vitro* growth medium, *defenseless*, showed similar growth as Col‐0 (Fig. 1b).

### Organelle ROS metabolism and signaling

One of the most sensitive assays to monitor the health status of plants is to measure the quantum yield of photosystem II, *F*_v_/*F*_m_ (Murchie & Lawson, 2013). In nonstressed, control plants, the *defenseless F*_v_/*F*_m_ values were similar to Col‐0 (Fig. 1c), indicating that as long as plants are maintained in clean growth conditions, they do not need active defenses. Plants use active production of apoplastic ROS for intra‐ and inter‐cellular signaling (Waszczak *et al*., 2018). We confirmed that *defenseless*, similarly to *rbohd*, lack activation of the apoplastic ROS burst, seen after treatment with the MAMP flg22 (Fig. S2).

Plant cells contain three genomes (nuclear, chloroplast, and mitochondrial), where signals need to be exchanged between organelles via anterograde and retrograde signaling for proper transcriptional regulation and plant function. Through applications of chemicals, the operation of these signals can be monitored as changes in *F*_v_/*F*_m_ (Shapiguzov & Kangasjärvi, 2022). Methyl viologen (MV) extracts electrons from photosystem I to create ROS in the chloroplasts, and antimycin A (AA) inhibits mitochondrial complex III activity leading to ROS production in the intermembrane space. With single treatments, this allows monitoring of the capacity of the chloroplast or mitochondria to withstand stress. In addition, the double MV + AA treatment also allows testing signaling and metabolic interactions between organelles, as the AA treatment will activate mitochondrial retrograde signaling to the nucleus, resulting in upregulation of mitochondrial *ALTERNATIVE OXIDASE1a* (*AOX1a*), which in turn will help to protect the chloroplasts from the ROS damage caused by the MV treatment (Shapiguzov *et al*., 2019). We used a previously established leaf disk assay, where the disks are incubated overnight in darkness with the single MV, AA, and double MV + AA treatments, followed by incubation in repeated cycles of blue light to activate ROS production from MV in the chloroplast (Shapiguzov & Kangasjärvi, 2022). Importantly, the overnight AA incubation allows increased *AOX1a* transcription to take place before the MV‐mediated ROS production is activated.

In Col‐0, MV alone induced strong damage, whereas AA treatment alone had minor effects (Fig. 1c). In line with previous results, the combined MV + AA treatment led to protection from the damage caused by MV (Fig. 1c; Shapiguzov *et al*., 2019). In *defenseless*, MV also caused damage, but this was not statistically significantly different from Col‐0, suggesting that the response to chloroplast ROS production is not influenced by the impaired defenses in *defenseless*. By contrast, the single AA treatment gave minor damage to *defenseless* (but not Col‐0). The combined MV + AA treatment also led to protection from ROS damage in *defenseless*, although to a lower level than in Col‐0. Overall, the single and double MV + AA assays suggest that *defenseless* has a wild‐type (WT) chloroplast ROS response and minor defects to mitochondrial stress responses.

To probe the contribution of other ROS sources, especially the peroxisome, we used the leaf disk assay with the catalase inhibitor 3‐AT (Gechev *et al*., 2002). Increasing concentrations of 3‐AT led to decreased *F*_v_/*F*_m_, indicating that improper ROS scavenging in the peroxisome feeds back into chloroplast function (Fig. S3a). In this assay, *defenseless* performed better than Col‐0. SA is not only activating defense signaling via its primary receptor NONEXPRESSER OF PR GENES 1 (Zavaliev & Dong, 2024), but SA also inhibits catalase to regulate the ROS balance during defense (Yuan *et al*., 2017). We tested a concentration series of SA in the leaf disk assay (Fig. S3b). At low concentration (0.1 mM), no difference between genotypes was observed, and at high concentration (0.5 mM), *F*_v_/*F*_m_ was severely decreased in both genotypes. The intermediate concentration (0.25 mM) was informative, where *defenseless* was more tolerant than Col‐0 (Fig. S3b).

Application of the air pollutant ozone that enters the plant through stomata allows the monitoring of ROS signaling via apoplast to nucleus (Xu *et al*., 2015). In our previous work, we used real‐time quantitative PCR (qPCR), microarrays, and RNA‐seq to investigate how apoplastic ROS signaling regulates transcriptional responses (Brosché *et al*., 2014). With qPCR, we tested seven different marker genes after 2‐h 350 nl l^−1^ ozone treatment (Figs 1d, S1b). *RAP2.6* (*RELATED to AP2 6*) expression was highly induced by ozone in Col‐0. By contrast, the *defenseless* mutant exhibited a significant reduction in *RAP2.6* expression compared with Col‐0 (Fig. 1d). However, within the *defenseless* background itself, ozone treatment still led to a significant upregulation of *RAP2.6* relative to its control, suggesting a partial response retained despite the impaired defenses. For *PHOSPHOLIPASE A 2A* and *CALMODULIN‐LIKE 37* (*CML37*), transcript levels were significantly elevated in response to ozone treatment in both genotypes. However, *CML37* was markedly upregulated in the *defenseless* mutant relative to Col‐0 under ozone treatment (Fig. 1d). Thus, we observed three different transcriptional ozone responses: lower, the same, and higher in *defenseless*. This suggests that the impaired signaling in *defenseless* can function as both positive and negative regulators of ROS transcriptional responses.

### The *defenseless* transcriptome

**Fig. 2:**
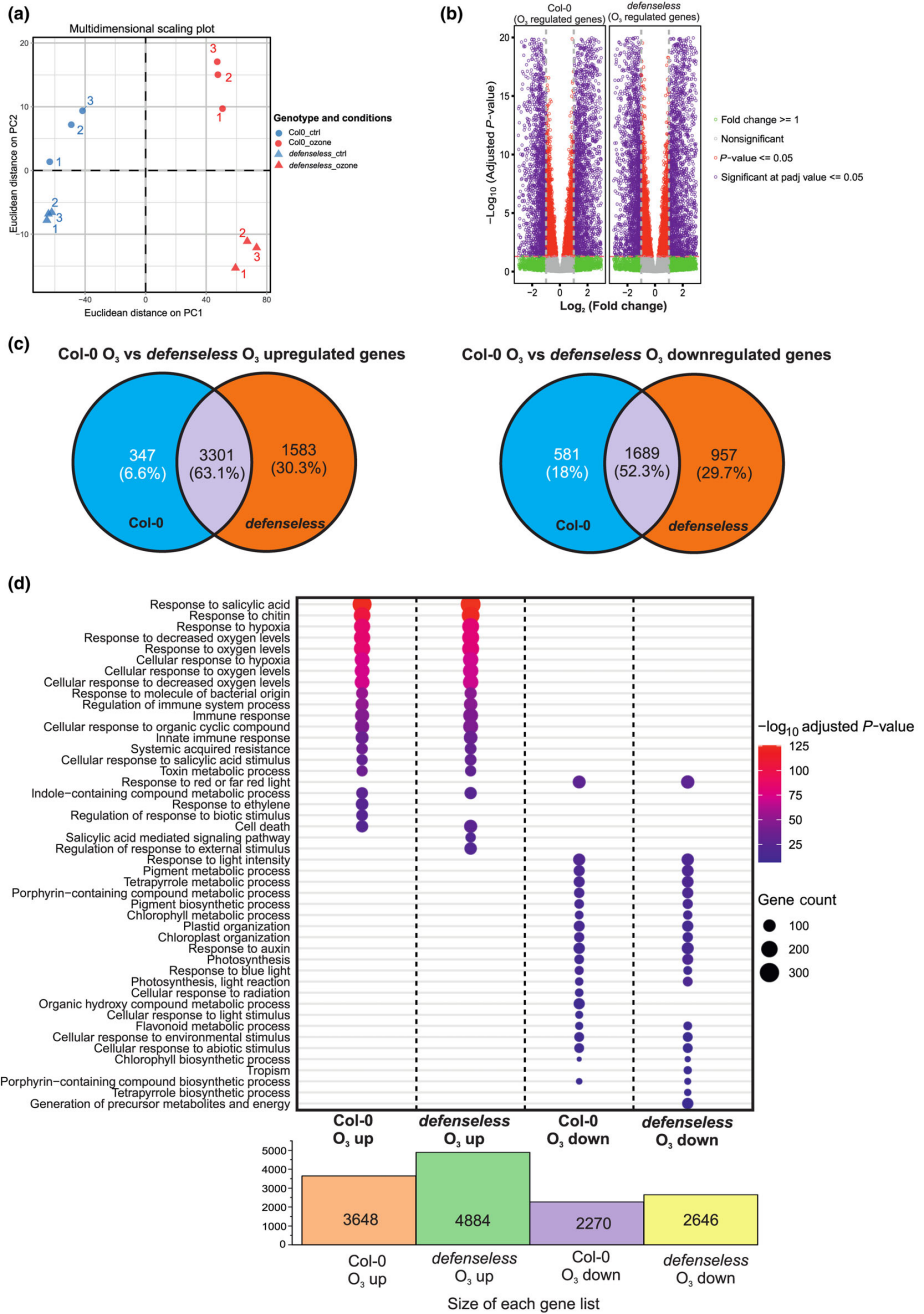
Transcriptional responses induced by ozone in Columbia‐0 (Col‐0) and *defenseless* mutant. Twenty‐two‐day‐old plants were exposed to 350 nl l^−1^ of O_3_ for 2 h, and transcriptome changes were accessed with RNA‐seq (*n* = 3). (a) Multidimensional scaling plot illustrating sample relationships based on transcriptome profiles. (b) Volcano plot illustrating the distribution of ozone‐regulated genes in Col‐0 and *defenseless*. Dots representing genes are color‐coded based on statistical significance and fold‐change thresholds: red dots indicate genes with *P*‐values < 0.05, green dots represent genes with log_2_FC ≥ 1, and purple dots highlight differentially expressed genes meeting both criteria. Gray dots correspond to nonsignificant genes. Upregulated genes are positioned on the right, while downregulated genes are on the left. (c) Venn diagrams of genes with significantly increased and decreased expression by ozone in Col‐0 and *defenseless*. (d) Dot plot of enriched Gene Ontology (GO) biological processes among ozone‐responsive genes. Bar diagram represents the gene list sizes used for the enrichment analysis depicted in the dot plot.

To gain full insights into which signaling is impaired in *defenseless* we performed RNA‐seq analysis after 2‐h ozone treatment. Multidimensional scaling (MDS) plots revealed distinct clustering of the samples (Fig. 2a). The ozone treatment was the main separator, followed by genotype. The separation by genotype was more pronounced during the ozone treatment than in control samples. We selected genes with significantly altered expression based on an adjusted *P*‐value < 0.05 and log_2_ fold change (< −1 for downregulated and > 1 for upregulated; Table S2). This filtering provided us with 3648 genes upregulated in Col‐0, 4884 genes upregulated in *defenseless*, 2270 genes downregulated in Col‐0, and 2646 genes downregulated in *defenseless*, which we displayed as volcano plots (Fig. 2b) and to get the differences between the genotypes they were compared in Venn diagrams (Fig. 2c). More than half of the differentially expressed genes were shared between the genotypes, suggesting that apoplastic ROS signaling is largely independent from hormone, EDS1, and RBOHD pathways.

To get the biological context of the differentially expressed genes, we performed Gene Ontology (GO) analysis on the GO category biological process. Again, *defenseless* displayed a remarkably similar profile as Col‐0, with only subtle differences; for example, ‘response to ethylene’ was upregulated by ozone in Col‐0 but not in *defenseless*.

**Fig. 3:**
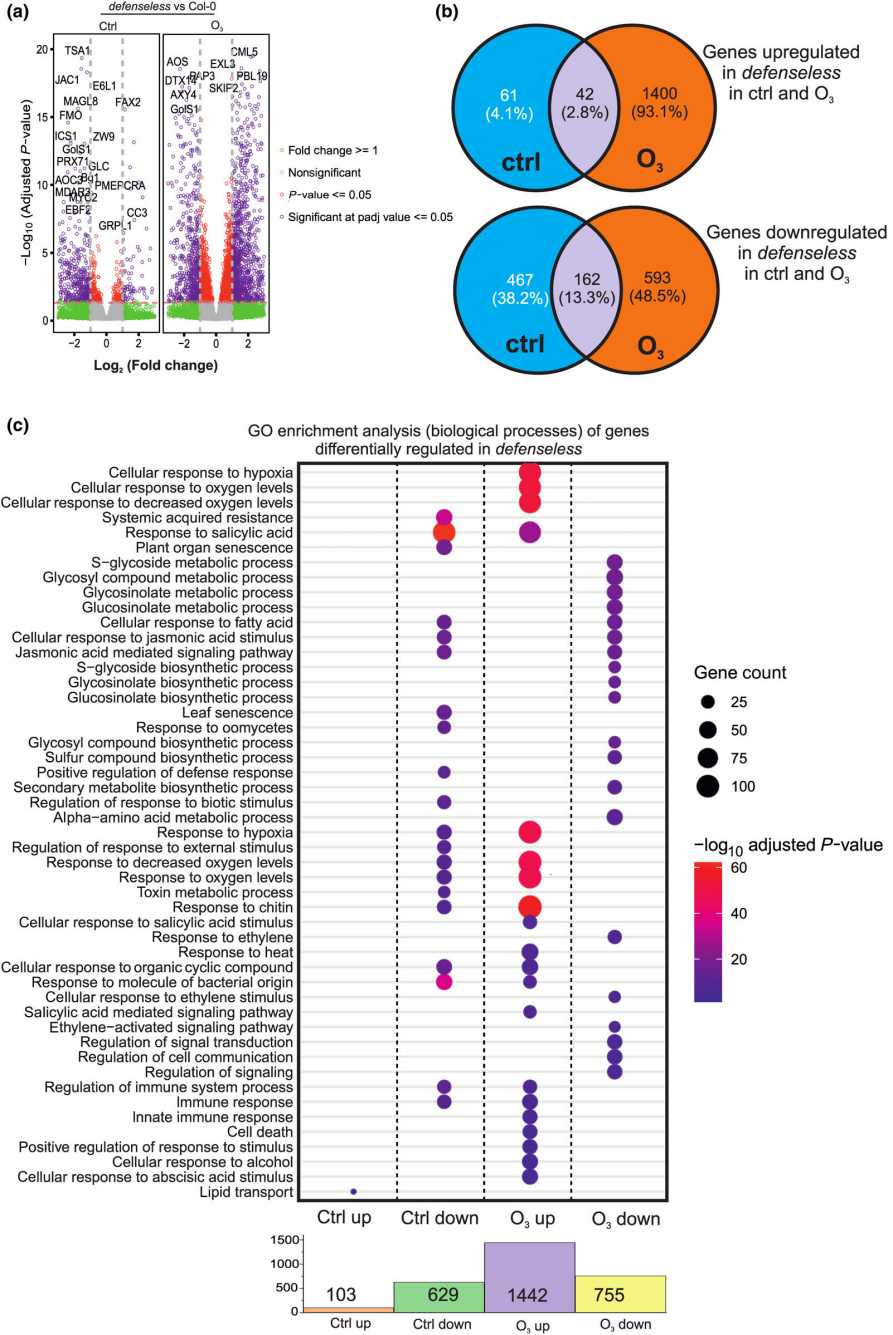
Difference between the Columbia‐0 (Col‐0) and *defenseless* transcriptome. (a) Volcano plots illustrating differentially expressed genes between Col‐0 and *defenseless* under control and ozone conditions. Upregulated genes appear on the right, downregulated on the left. Dots are color‐coded by statistical and fold‐change thresholds: red (*P* < 0.05), green (log_2_FC ≥ 1), purple (differentially expressed genes meeting both criteria with gene labels), and gray (nonsignificant). (b) Venn diagrams displaying the genotype difference between the Col‐0 and *defenseless* differentially expressed genes under control and ozone conditions. These gene sets were used for subsequent enrichment analyses. (c) Dot plot of enriched Gene Ontology (GO) biological processes among differentially expressed genes in *defenseless* under control and ozone treatments. The accompanying bar chart indicates gene set sizes used for enrichment analysis.

The analysis above was based on the identification of genes significantly differentially expressed in Col‐0 and *defenseless* through analysis of control vs ozone samples. The complementary approach is to instead analyze Col‐0 vs *defenseless* control samples, and Col‐0 vs *defenseless* ozone samples (Fig. 3; Table S3). We used the same selection criteria as above (adjusted *P*‐value < 0.05 and log_2_ fold change < −1 for downregulated and > 1 for upregulated) and plotted the genes as a volcano plot (Fig. 3a) and in Venn diagrams (Fig. 3b). We identified 103 genes with higher expression in *defenseless* in control, 629 genes with lower expression in *defenseless* in control, 1442 genes with higher expression in *defenseless* in ozone and 755 genes with lower expression in *defenseless* in ozone. As already suggested by the MDS plot (Fig. 2a), the *defenseless* mutant had more differentially expressed genes in the ozone treatment than in control, and with the most genes belonging to the category upregulated in *defenseless* in the ozone treatment (Fig. 3b). This matches the profile of the *CML37* marker gene used in qPCR (Fig. 1d). To put the transcriptional differences between Col‐0 and *defenseless* into a biological context we performed GO analysis for biological process (Fig. 3c). As anticipated based on the impaired signaling pathways in *defenseless*, many GO categories related to hormones and defenses showed enrichment among the genes differentially expressed in *defenseless*.

In control conditions, the biological process lipid transport was upregulated in *defenseless*, whereas genes associated with SA signaling, systemic acquired resistance, and bacterial molecular recognition were downregulated in *defenseless*. Under ozone treatment, there was a significant upregulation of genes involved in cellular response to hypoxia, oxygen homeostasis, chitin response, and SA‐mediated signaling pathways, indicating a broad activation of stress‐responsive pathways. Conversely, genes implicated in S‐glycoside and glucosinolate biosynthesis, ET signaling, signal transduction regulation, and cell communication were downregulated in *defenseless*. Plants manage ROS levels via antioxidants and enzymes that scavenge ROS, for example catalase that removes H_2_O_2_. In Arabidopsis, at least 150 genes are involved in determining the ROS levels (Mittler *et al*., 2004) and we checked their expression levels (Table S4). In control treatment, few genes differed between Col‐0 and *defenseless*. In ozone, more genes had differential expression between *defenseless* and Col‐0, but for many of these, the expression was higher in *defenseless*, suggesting that the mutant would have increased capacity to handle ROS. We continued to measure reduced and oxidized ascorbic acid, where *defenseless* had increased amount of reduced ascorbic acid (Fig. S4).

**Fig. 4:**
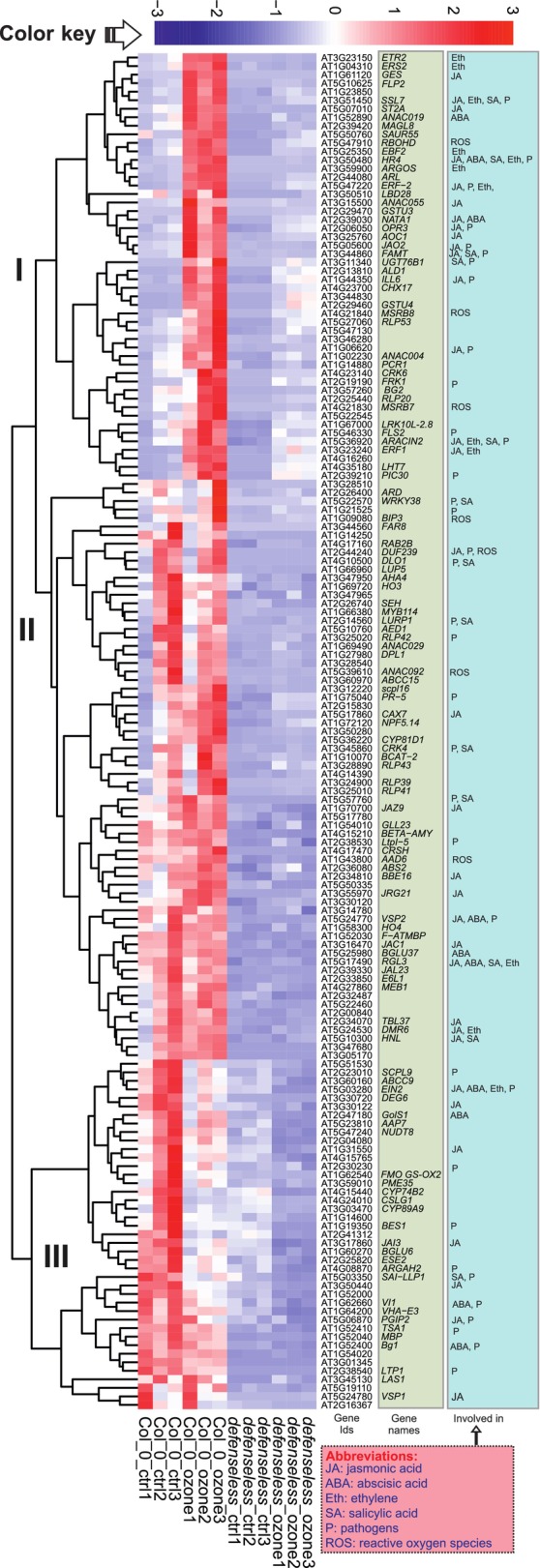
Heatmap of the 162 genes downregulated in the *defenseless* mutant under both control and ozone conditions. The first column lists gene accession numbers, the second lists gene symbols (retrieved from the TAIR database), and the third indicates associated functional pathways (see also Supporting Information Table S5). ABA, abscisic acid; Eth, ethylene; JA, jasmonic acid; P, pathogens; ROS, reactive oxygen species; SA, salicylic acid.

To identify a core set of genes with decreased expression in *defenseless* and their biological context, we selected 162 genes that were significantly downregulated in *defenseless* in both control and ozone treatment (Fig. 3b), and performed hierarchical clustering using Euclidean distance and complete linkage (Fig. 4). Cluster I contained genes strongly upregulated by ozone in Col‐0 but not in *defenseless*, Cluster II had genes that have high expression in Col‐0 (both control and ozone) and low expression in *defenseless*, Cluster III had genes with high expression in Col‐0 control and low expression in *defenseless*. In this core set of genes, there are numerous regulators of hormone and pathogen signaling that have been extensively studied for their function in plant–pathogen interaction, for example the *FLAGELLIN‐SENSITIVE 2 (FLS2)* receptor that detects flagellin (Chinchilla *et al*., 2006), *FLG22‐INDUCED RECEPTOR‐LIKE KINASE 1*, which has been used as a marker gene for pathogen signaling (Asai *et al*., 2002), and *AGD2‐LIKE DEFENSE RESPONSE PROTEIN 1* involved in the pipecolic acid biosynthetic pathway that is a key signal for systemic acquired resistance (Bernsdorff *et al*., 2016). Strikingly, there are also multiple genes from JA biosynthesis or catabolism (*ALLENE OXIDE CYCLASE 1 (AOC1)*, *OXOPHYTODIENOATE‐REDUCTASE 3 (OPR3)*, and *JASMONIC ACID OXIDASE 2 (JAO2)*) and the ET signaling pathway (*ETHYLENE RESPONSE 2 (ETR2)*, *ETHYLENE RESPONSE SENSOR 2 (ERS2)*, and *EIN3‐BINDING F BOX PROTEIN 2 (EBF2)*). Overall, this shows that *defenseless* is impaired in the signaling pathways that would be expected from the mutants used to create *defenseless*.

### The response to pathogens in *defenseless*

**Fig. 5:**
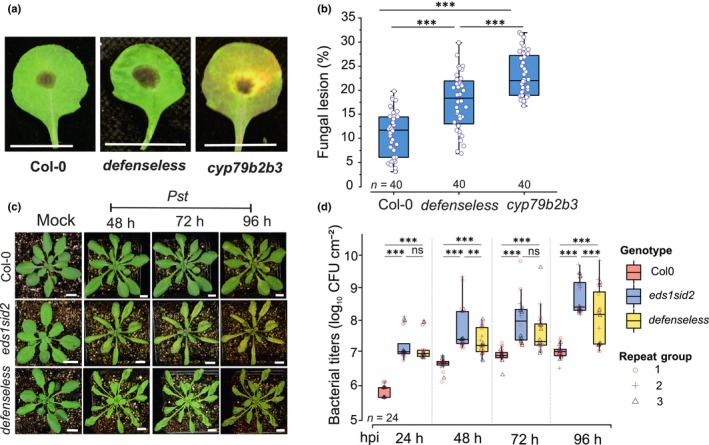
Pathogen infection assays of Columbia‐0 (Col‐0) and different mutants (*defenseless*, *cyp79b2b3*, and *eds1 sid2*) by fungal and bacterial pathogens. (a) *Alternaria alternata* infection in Col‐0, *defenseless*, and *cyp79b2b3* (positive control). Representative images show necrotic lesions indicative of fungal colonization. (b) The lesion area was quantified as a percentage of the total leaf surface. Each genotype included 40 biological leaves (*n* = 40). (c) *Pseudomonas syringae* pv *tomato* DC3000 (*Pst*) infection in Col‐0, *defenseless*, and *eds1sid2*. (d) A time‐course analysis measured bacterial titers at 24‐, 48‐, 72‐, and 96‐h postinoculation (hpi). Individual measurements from 40 biological replicates (b) and 24 measurements across three repeat groups (d) are displayed as box‐and‐whisker plots. Boxes denote the interquartile range (25^th^–75^th^ percentiles) with the median indicated by a horizontal line. Circles (and distinct symbols for repeat groups) represent individual data points, and whiskers indicate the minimum and maximum values. Statistical significance was assessed using one‐way ANOVA followed by Tukey's honest significant difference (HSD) test. Significance levels: ***, *P* < 0.0001; **, *P* < 0.05; ns, not significant (*P* > 0.05). Bacterial assays were conducted with 24 biological replicates across three independent experiments (*n* = 8 per replicate).

**Fig. 6:**
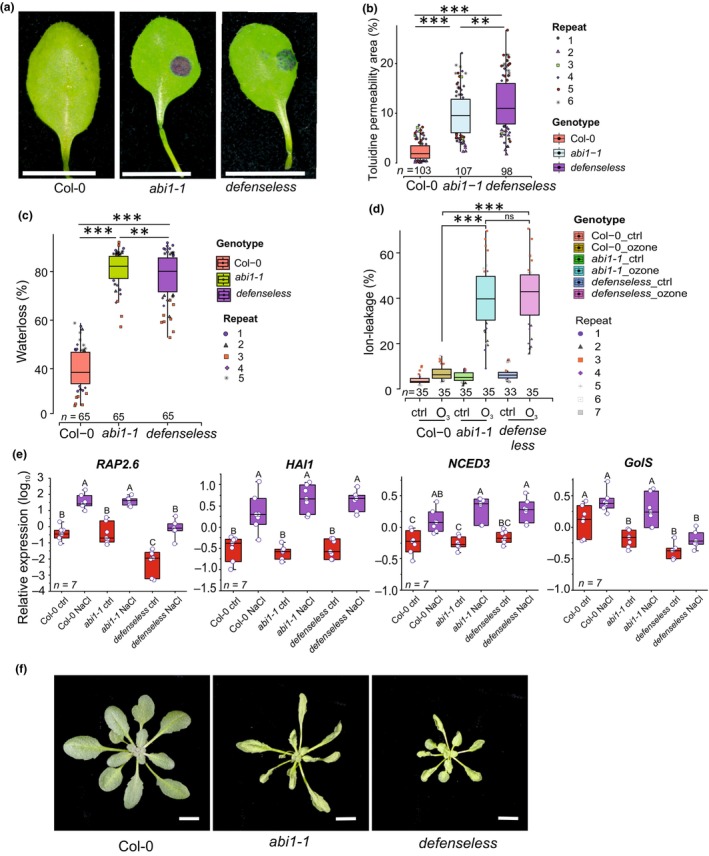
Developmental and abiotic stress assays in Columbia‐0 (Col‐0) and mutants (*abi1‐1* and *defenseless*). (a) Toluidine blue (TB) staining of leaves following 2‐h incubation. Col‐0 showed limited staining, indicative of an intact cuticle, while *abi1‐1* and *defenseless* displayed strong blue staining, consistent with cuticular defects. (b) Quantification of TB permeability revealed significant differences between *abi1‐1* and *defenseless* (*P* < 0.05), and a highly significant difference between Col‐0 and both mutants (*P* < 0.01). Data were analyzed using one‐way ANOVA. (c) Water loss (%) across genotypes (*n* = 65, 5 biological repeat groups). One‐way ANOVA with Tukey's honest significant difference (HSD) test indicated highly significant water loss in *abi1‐1* and *defenseless* compared with Col‐0 (*P* < 0.01), and a significant difference between the two mutants (*P* < 0.05). (d) Ion leakage as a measure of ozone‐induced cell death following 6‐h O_3_ exposure. Box plots represent data from seven biological repeats (*n* = 33–35 plants). Two‐way ANOVA with Tukey's HSD test showed highly significant differences (*P* < 0.01) between Col‐0 and both mutants under ozone treatment, while no significant difference (ns, *P* > 0.05) was observed between *abi1‐1* and *defenseless*. (e) Reverse transcription quantitative polymerase chain reaction (RT‐qPCR) was used to assess the relative expression of marker genes (*RAP2.6*, *HAI1*, *NCED3*, and *GolS*) under control and NaCl (see Supporting Information Fig. S5d for additional genes). Data represent seven biological replicates, and statistical significance was determined using two‐way ANOVA, followed by Tukey's test (*P* < 0.05). Samples with different letters are significantly different. (f) Representative plant images following 2‐h ozone exposure. Bars, 1 cm.

To test *defenseless* in plant–pathogen interaction, we performed assays with both fungal and bacterial pathogens (Fig. 5). After 1 wk, fungal lesions were quantified in Col‐0 and *defenseless*, and in *cyp79b2b3* (defective in indolic glucosinolates biosynthesis) used as positive control (Fig. 5a). Fungal lesion areas were significantly larger in *defenseless* mutants compared with Col‐0 WT (*P* < 0.01; Fig. 5b), but the lesion area of infection of the *defenseless* mutant was less than that of the *cyp79b2b3* mutant. Here, it should be noted that the *abi1‐1* mutation provides a permeable cuticle (Fig. 6), which was previously shown to give strong resistance toward fungal pathogens (Bessire *et al*., 2007). As *defenseless* was susceptible to *Alternaria* infection, this suggests that potential resistance from a permeable cuticle is not enough to overcome the other impaired defenses in *defenseless*.

For bacterial pathogen assays, we used spray inoculation with *Pseudomonas syringae* pv *tomato* DC3000. In addition to *defenseless*, we also used the *eds1 sid2* double mutant, as SA is considered the main defense against bacterial pathogens. Both *eds1 sid2* and *defenseless* exhibited significantly higher susceptibility to bacterial infection relative to Col‐0. Visible disease symptoms (chlorosis, necrosis, and tissue collapse) became apparent after 48 h postinoculation (hpi; Fig. 5c). Quantitative bacterial growth assays revealed markedly elevated bacterial titers as early as 24 hpi, with statistically significant differences persisting through 96 hpi (Fig. 5d). While both *eds1 sid2* and *defenseless* were highly susceptible, the *eds1 sid2* double mutant was the most susceptible mutant. This suggests that other mutations in *defenseless* can provide some level of resistance, for example, the *abi1‐1* mutation that not only provides a permeable cuticle but also leads to more open stomata (Merilo *et al*., 2013), which alters the aquatic environment inside of the leaf and hence bacterial proliferation (Lajeunesse *et al*., 2023).

### The response to abiotic stress in *defenseless*

ABA is considered the main hormone in various signaling pathways related to abiotic stress. In addition, ABA is also a regulator of developmental responses, for example, seed germination and cuticle formation (Cui *et al*., 2016). To check for cuticle permeability, we used toluidine blue stain in *abi1‐1* and *defenseless* (Fig. 6a). Quantitative analysis of the toluidine‐stained surface area revealed increased cuticle permeability in both mutants, with a subtle increase in *defenseless* vs *abi1‐1*. This suggests that ABA signaling is the main hormone in regulating cuticle formation, with a limited contribution from other hormone signaling pathways impaired in *defenseless*.

ABA is also the main regulator of guard cell function in the opening and closing of stomata. This impacts many stresses, including the response to bacterial pathogens, entry of ozone into the plant, and water balance. A classical assay for guard cell function is to measure water loss from detached leaves (Klein *et al*., 1996; Outlaw Jr, 2003), but here it should be considered that this assay will not distinguish between water that is lost through permeable cuticle vs open stomata, as both will contribute to the water that evaporates from leaves. Both *abi1‐1* and *defenseless* mutants demonstrated significantly higher water loss compared with Col‐0. While there was a statistically significant change between *abi1‐1* and *defenseless*, the difference in water loss was so minor that it is probably not biologically relevant. Thus, for both cuticle permeability and water loss, it is the *abi1‐1* mutation in *defenseless* that acts as the main contributor to the *defenseless* phenotype.

We use treatments with the air pollutant ozone not only to study the role of apoplastic ROS in activation of defense signaling but also to study how ROS regulates cell death (Brosché *et al*., 2014). We quantified cell death after a 6‐h exposure to ozone, in *abi1‐1* and *defenseless* (Fig. 6d). As mutants with open stomata and/or permeable cuticle are highly ozone sensitive due to increased amount of ozone entering the plant, we predicted that the *abi1‐1* mutant would be the main source of ozone sensitivity in the *defenseless* mutant. Both mutants were highly ozone sensitive (Fig. 6d,f), but with no significant difference between the mutants, suggesting that *abi1‐1* provides the high ozone sensitivity to *defenseless*.

Another abiotic stress that uses ABA as a signaling intermediate is NaCl stress. We first tested *defenseless* in a NaCl root growth assay, where root elongation was observed at increasing concentrations of NaCl. No difference in root growth was observed between Col‐0 and *defenseless* (Fig. S5d). To study transcriptional responses to NaCl in Arabidopsis research, it is common to use relatively young seedlings. As we wanted to stay consistent in our use of adult plants for all transcript profiling assays, we performed the assay by putting a 10 μl drop of 150 mM NaCl on the surface of fully developed leaves. Samples were harvested after 6 h and from corresponding control samples treated with water. We selected seven marker genes for qPCR (Figs 6e, S5d), based on a recent NaCl transcriptome study (Lamers *et al*., 2025), as well as marker gene and transcriptome data from the ozone experiments (Figs 1, 2, 3, 4). As in the ozone treatment (Fig. 1d), increased transcript levels were seen after NaCl treatment for *RAP2.6* (Fig. 6e). Notably, *RAP2.6* expression was decreased in *defenseless* (but not in *abi1‐1*), again consistent with the data from the ozone treatment (Fig. 1e). Also, *ERF109* showed the same profile (Fig. S5d). *HIGHLY ABA‐INDUCED PP2C GENE 1 (HAI1*) and *RESPONSIVE TO DESICCATION 20* were induced in response to NaCl stress in all three genotypes (Figs 6e, S5d). Similarly, *NCED3* (9‐*cis*‐Epoxycarotenoid Dioxygenase 3), a key rate‐limiting enzyme in the ABA biosynthesis pathway, was also significantly upregulated under salt stress in all genotypes. One of the few genes associated with ABA signaling in the core 162 genes downregulated in *defenseless* was *Gol‐S* (Galactinol synthase; Fig. 4). *GolS* is involved in the biosynthesis of galactinol, a precursor of raffinose family oligosaccharides important for osmotic and oxidative stress tolerance. Consistent with our transcriptome data, transcript levels for Gol‐S were low in *defenseless*. However, it was significantly upregulated by NaCl in the *abi1‐1* mutant. Overall, the *defenseless* mutant was more impaired in NaCl transcriptional responses than the *abi1‐1* mutant, suggesting contributions from other hormones than ABA to signaling in the response to NaCl.

## Discussion

In this work, we investigated whether it is possible to generate a plant that lacks all the major plant defense signaling pathways, and how this plant would respond to perturbations of organellar function, abiotic and biotic stress. We selected mutants impaired in hormone signaling or biosynthesis (ABA – *abi1‐1*, JA – *coi1‐16*, ET – *ein2*, and SA – *sid2*). In pathogen signaling, EDS1 is a key node in basal and ETI (Cui *et al*., 2017), and ROS generated from RBOHD act as signaling molecules in both abiotic and biotic stress (Miller *et al*., 2009). In *defenseless* (*abi1‐1 coi1‐16 eds1 ein2 rbohd sid2*), the majority of the well‐characterized plant defense signals should be removed. The rationale for choosing these mutants was also based on previous work with double, triple, and quadruple mutants used to study defense signaling. Characterization of *eds1 sid2* showed that EDS1 and SA act redundantly in pathogen defenses (Venugopal *et al*., 2009; Cui *et al*., 2017). Extensive work with *dde2 ein2 pad4 sid2*, deficient in JA biosynthesis (*dde2*, a mutation in *aos*), ET signaling, SA biosynthesis, and for PAD4 (an interacting partner protein to EDS1) showed that using single mutants is not enough to understand defense signaling: when one signal pathway is missing other signals can compensate to still provide defense (Hillmer *et al*., 2017). We have previously also analyzed the transcriptional responses of many of the single (*abi1‐1*, *coi1‐16*, *ein2*, and *sid2*), double (*coi1 sid2*, *coi1 ein2*, and *ein2 sid2*), and triple mutants (*coi1 ein2 sid2*) that are part of *defenseless* (Xu *et al*., 2015; Vuorinen *et al*., 2021), and these mutants have a mostly intact transcriptional response. Hence, *defenseless* should be a valuable complement to single mutant studies to get a broader view of the redundancy in signaling. With the *defenseless* mutant, we were able to evaluate the contribution of hormone and pathogen signaling pathways to defense and defense signaling. As a large part of transcriptional regulation appears to be intact in *defenseless*, this suggests that additional signaling pathways contribute to plant defense and defense signaling, via for example Ca^2+^ or kinases.

### Transcriptional regulation in *defenseless*

We used treatment with the air pollutant ozone to initiate ROS signaling from the apoplast (Xu *et al*., 2015; Waszczak *et al*., 2018). In line with our previous results (Xu *et al*., 2015), several thousand genes were up‐ and downregulated by the ozone treatment in both Col‐0 and in *defenseless* (Fig. 2). The large number of ozone‐regulated genes in *defenseless* suggests that there are alternative signaling pathways that operate in parallel with hormone signaling. These independent signaling pathways could, for example, be connected to Ca^2+^, an important signaling molecule in many stress signaling pathways (Luan & Wang, 2021), or to kinase‐mediated signaling, for example, via mitogen‐activated protein (MAP) kinases that are activated in response to many stresses, including ozone (Ahlfors *et al*., 2004; Sun & Zhang, 2022). In this scenario, ozone initiates ROS signaling in the apoplast, leading to activation of Ca^2+^ channels and/or activation of kinase signaling that relays the signal to activation of transcription factors in the nucleus and transcriptional reprogramming.

However, even if there is a large overlap in ozone‐regulated genes between WT and *defenseless*, there are numerous genes that show altered expression in the mutant. The largest category of *defenseless* differentially expressed genes included genes with increased expression in the ozone‐treated mutant. This can be seen for the marker gene *CML37* in qPCR (Fig. 1d), and in transcriptome data with 1442 genes (Fig. 3). GO analysis of these genes suggests functions related to oxygen sensing, SA, and immunity signaling. As these genes have higher expression in *defenseless*, it suggests that hormone signaling acts as a negative regulator to keep their expression levels low. This is in line with studies of autoimmune mutants, where too strong activation of plant defenses leads to impaired growth (Chakraborty *et al*., 2018). Thus, defense signaling in plants should balance appropriate activation of defense with overactivation that could lead to detrimental effects. The other category of genes with differential expression are genes downregulated in *defenseless*, in control, and/or ozone treatment. GO enrichment of these genes indicated their role in SA, JA, ET, and pathogen/defense signaling, and that these genes are downregulated in *defenseless* is consistent with this mutant being impaired in hormone and defense signaling (Figs 3, 4).

ROS functions as a crucial secondary messenger in diverse plant stress responses, including plant–pathogen interactions. Previous work by Vuorinen *et al*. (2021) demonstrated a substantial overlap between genes transcriptionally regulated by ozone exposure and those responsive to *Botrytis cinerea* infection, suggesting a shared transcriptional signature indicative of ROS‐mediated signaling. To further investigate the relevance of ozone‐induced transcriptional changes as a readout for ROS‐associated plant responses (Vaahtera *et al*., 2014), we used data from the Genevestigator database to evaluate transcriptional regulation of four marker genes we used for qPCR. This revealed that strongly ozone‐responsive genes (*CML37*, *CRK37*, *MDHAR*, and *RAP2.6*) were consistently and similarly regulated by multiple biotic stress conditions, including infections by bacterial and fungal pathogens (Fig. S6). This supports the notion that ozone initiates apoplastic ROS signaling (Waszczak *et al*., 2018), similarly to the ROS signaling activated during pathogen attack. Consequently, our ozone RNA‐seq dataset provides a useful resource for dissecting ROS‐dependent regulatory networks involved in plant immune responses.

To get a further biological context of the genes downregulated in *defenseless*, we focused on the core 162 genes downregulated in *defenseless* in both control and ozone treatment (Fig. 4). Among these core genes are many genes that belong to JA biosynthesis/signaling (*AOC1*, *OPR3*, *JAO2*, and *JAZ9*), ET signaling (*ETR2*, *ERS2*, and *EBF2*), and transcription factors characterized as executors of ET/JA signaling (*ERF1*; Lorenzo *et al*., 2003) and SA signaling (*WRKY38*; Kim *et al*., 2008). Overall, the *defenseless* transcriptome analysis indicates that the mutant is severely impaired in specific aspects of JA/SA/ET/defense signaling, but at the same time the mutant also has other signaling pathways that are functional to mediate the ROS signal into transcriptional changes.

The transcription factor *RAP2.6* used as a marker gene in qPCR illustrates another aspect of signaling to take into consideration (Figs 1d, 6e). Expression of *RAP2.6* is regulated by several biotic and abiotic stresses, and by JA and ABA signaling (Zhu *et al*., 2010; Vuorinen *et al*., 2021). In *defenseless*, its expression levels were very low, but both ozone and salt stress led to increased transcript levels in *defenseless*. This indicates that many different signaling pathways can target this gene, both the hormone‐dependent and independent signaling pathways.

### Stress responses in *defenseless*

We established the *defenseless* mutant as a platform to study defense and hormone signaling. However, the transcriptional response is only one aspect of defense, and to get a broader view of the *defenseless* phenotype, we evaluated its stress and developmental phenotypes. When grown *in vitro* or in a clean growth room or growth chambers, the *defenseless* mutant does not appear to be visibly stressed, and its *F*_v_/*F*_m_ value is the same as WT, suggesting that as long as it is maintained in nonstressful growth conditions, it performs well. However, *defenseless* is smaller than Col‐0 (Fig. S1a). This is likely a reflection of hormone regulation of growth. For example, it has long been observed that ABA biosynthesis and signaling mutants are smaller than WT (Cutler *et al*., 2010).

ROS are used as signal molecules in many stress signaling pathways, but their location differs according to stress; for example, ozone and pathogens initiate ROS signaling from the apoplast (Waszczak *et al*., 2018). To investigate organellar (chloroplast, mitochondria, and peroxisome) ROS production, we used chemicals to perturb the function in chloroplast (MV), mitochondria (AA), and peroxisome (3‐AT). In the single MV treatment, this showed similar damage in WT and *defenseless*, indicating that impaired signaling in *defenseless* is not contributing to chloroplast function (Fig. 1c). By contrast, the *defenseless* mutant was sensitive to the AA treatment, which initiates ROS production in mitochondria through inhibition of the electron transport chain. Proper exchange of signals between chloroplast, mitochondria, and nucleus is needed to protect against stress that affects the organelles. This can be evaluated in a combined MV + AA assay, where signaling initiated from AA stressed mitochondria goes to the nucleus, resulting in transcriptional reprogramming that ultimately helps the chloroplast to better tolerate MV (Shapiguzov *et al*., 2019). The combined MV + AA treatment gave protection toward MV damage both in WT and *defenseless*, but to a slightly lower level in *defenseless*. Application of the catalase inhibitor 3‐AT showed increased tolerance in *defenseless* (Fig. S3a). Overall, the 3‐AT, MV, and AA assays suggest a slightly altered response from the chemical that impairs mitochondria function, but, as *defenseless* can still execute protection for the chloroplast, this suggests a mostly intact organellar signaling network in *defenseless*.

Most of the previous work to impair multiple signaling pathways has focused on ET, JA, SA, and pathogen signaling (Xu *et al*., 2015; Hillmer *et al*., 2017). In *defenseless*, we also impaired ABA signaling with the *abi1‐1* mutant. To further evaluate *defenseless*, we performed a series of assays where ABA is considered the main regulatory hormone, notably cuticle development and regulation of stomatal aperture. The plant cuticle constitutes a critical barrier limiting pathogen entry. Both *abi1‐1* and *defenseless* mutants exhibited significantly increased toluidine blue uptake relative to Col‐0, indicating compromised cuticular integrity (Fig. 6). Water loss from detached leaves acts as an indicator for both permeable cuticle and open stomata, as these are the main places where water can evaporate from leaves. Again, both *abi1‐1* and *defenseless* showed high water loss compared with Col‐0 (Fig. 6).

To probe *defenseless* responses in defense against pathogens, we used both fungal and bacterial pathogens. The lesions observed following *Alternaria* (a necrotrophic pathogen) infection directly implicate compromised antifungal defenses, likely due to attenuated JA‐ and ET‐mediated signaling pathways (Qi *et al*., 2012). As a positive control, we employed the *Arabidopsis* glucosinolate biosynthesis mutant *cyp79b2b3*, which had larger lesions than the *defenseless* mutants (Fig. 5a,b). Interpretation of *defenseless* fungal defenses is complicated by the permeable cuticle observed in *defenseless* (Fig. 6). A permeable cuticle, including that seen in strongly impaired ABA mutants, provides strong resistance to fungal pathogens (Serrano *et al*., 2014). However, as *defenseless* was susceptible to *Alternaria* infection, this suggests that the resistance provided by a permeable cuticle was insufficient to overcome the other impaired defenses in *defenseless*.

Both *defenseless* and the *eds1 sid2* double mutant showed high susceptibility to the bacterial pathogen *Pst*. However, *defenseless* was not more susceptible than *eds1 sid2*. As SA is considered the main defense hormone, this implicates SA‐mediated transcriptional activation as the main reason for susceptibility in *defenseless*. Also, the interpretation of bacterial susceptibility is challenging. As *defenseless* has a permeable cuticle and more open stomata from the *abi1‐1* mutation, this allows additional entry points for the bacteria. In addition, mutants with more open stomata are more resistant to *Pst* infection due to loss of water, preventing the formation of an aqueous phase required for *Pst* growth (Kemppinen *et al*., 2025). As *defenseless* was susceptible to *Pst* to almost the same level as *eds1 sid2*, this suggests that even if these other mechanisms may have an impact, they are not enough to override the loss of SA and EDS1 in defense against *Pst*.

Defense against damage from ozone relies on several components, where the first barrier consists of preventing ozone entry into the plants. Mutants with more open stomata, permeable cuticle, or other epidermal defects are ozone sensitive due to higher levels of ozone entering the plant (Waszczak *et al*., 2024). Once inside of the plant, the balance of hormone signaling influences to which extent ozone will give tissue damage through activation of programmed cell death (Xu *et al*., 2015). In both *abi1‐1* and *defenseless*, there was extensive tissue damage in leaves, which was further supported by quantification of the damage with ion leakage (Fig. 6). As the damage was at similar levels in both *abi1‐1* and *defenseless*, this suggests that very high entry of ozone into both mutants via the permeable cuticle and open stomata caused the damage and other signaling pathways did not have a major contribution.

NaCl acts as a stress in plants via ABA‐dependent and ABA‐independent signaling pathways (Yoshida *et al*., 2014). We employed two different assays to evaluate *defenseless* responses to NaCl, root growth inhibition, and transcriptional responses. Root growth was similar in Col‐0 and *defenseless* in increasing concentrations of NaCl (Fig. S5b,c). In qPCR, we used several marker genes, including ABA biosynthesis (*NCED3*), ABA signaling (*HAI1*), transcription factors (*RAP2.6*, *ERF109*), and osmoprotectant biosynthesis (*GolS*). Both *HAI1* and *NCED3*, considered as markers for ABA signaling (Huang *et al*., 2018), were similarly induced in all three genotypes, suggesting that their regulation by NaCl is through an ABA‐independent signaling pathway. By contrast, increased expression by NaCl for *RAP2.6* and *ERF109* was impaired only in *defenseless* (and not in *abi1‐1*). This suggests that it is not impaired ABA signaling in *defenseless* that leads to their lower expression, and instead it's another signaling pathway. This is likely to be JA, as both genes are also suggested to be JA‐regulated (Cai *et al*., 2014; Vuorinen *et al*., 2021). We selected *Gol‐S* as a marker gene as it belonged to the core 162 genes downregulated in *defenseless* (Fig. 4). Consistent with the transcriptome data, *Gol‐S* had decreased transcript levels in *defenseless*. However, it was not downregulated in *abi1‐1*, suggesting that similarly to *RAP2.6* and *ERF109*, an ABA‐independent signaling pathway is activated by the NaCl treatment.

### Which defense signals and plant defenses remain in *defenseless*?

One intriguing observation was the occasional collapse of *defenseless* in clean good growth conditions (Fig. 1b). This suggests that even if most of the plants show no signs of stress as indicated by *F*_v_/*F*_m_ (Fig. 1c), the lack of defenses makes *defenseless* very susceptible to stress once it is initiated. However, there are many nuances to consider; *defenseless* retained some level of resistance to both *A. alternata* and *Pst* DC3000. While susceptibility was elevated relative to Col‐0, it remained lower than that observed in positive control mutants deficient in specific, individual defense components. Transcriptomic profiling via RNA‐seq further revealed that relatively few genes were differentially expressed in both Col‐0 and *defenseless* mutants, indicating that the overall transcriptional response to ozone remains largely intact. These findings suggest that complete removal of defense signaling requires simultaneous inactivation of multiple, potentially redundant defense mechanisms. The challenge of testing other additional signaling components, for example, Ca^2+^ signaling or MAP kinase signaling, comes from large genetic redundancy in both Ca^2+^ channels and the MAP kinase signaling cascade (Luan & Wang, 2021; Sun & Zhang, 2022). Applications of specific chemical inhibitors, for example Ca^2+^ channel inhibitors or chelators, in the *defenseless* mutant could reveal which other signaling pathways are still active.

Plant defenses rely not only on signaling once the stress is detected but also on many different types of defenses via antioxidants and scavengers to manage ROS levels, secondary metabolites, and barriers (cell wall, cuticle, callose, suberin deposition, trichomes, etc.). Here, *defenseless* showed both impairments, for example a permeable cuticle (Fig. 6a), but at the same time an intact or possibly enhanced defense, for example an increase in reduced ascorbate and better tolerance to catalase inhibitors. To make a true *defenseless* mutant, we will likely need to combine mutations that not only impair signaling but also those that disrupt various preformed or metabolite‐based defenses, such as *pad3* impaired in CYP71B15 (Cytochrome P450 71B15) that catalyze the last step in camalexin biosynthesis (Schuhegger *et al*., 2006) or *pad2* impaired in glutamate‐cysteine ligase that catalyze the rate‐limiting step of glutathione biosynthesis (Parisy *et al*., 2007).

Collectively, our data underscore the complexity and robustness of the plant defense signaling network and provide a foundation for future studies aimed at disentangling the interplay and relative contributions of discrete signaling pathways within a combinatorial framework.

## Competing interests

None declared.

## Author contributions

BB and MB performed the experiments. BB analyzed the RNA‐seq data. MB generated the *defenseless* mutant and designed the project. BB and MB wrote the manuscript.

## Disclaimer

The New Phytologist Foundation remains neutral with regard to jurisdictional claims in maps and in any institutional affiliations.

## Supporting information


**Fig. S1** Growth and stress responses in *defenseless*.
**Fig. S2** Flg22 induced ROS burst in Col‐0, *defenseless* and *rbohD* from three biological repeats, each consisting of 24 leaf disks.
**Fig. S3** Leaf disk assay of Col‐0 and *defenseless* under treatments with 3‐AT (a) or SA (b).
**Fig. S4** Ascorbate redox status in Col‐0 and *defenseless* under control conditions.
**Fig. S5** Cuticle and NaCl responses in *defenseless*.
**Fig. S6** Transcriptomic profiles of selected genes induced under various treatment conditions, extracted from the Genevestigator Plants database.
**Methods S1** Experimental details and materials and methods for transcriptome analysis, abiotic and biotic stress assays.

**Table S1** Primer sequences for genotyping and RT‐qPCR, along with references.

**Table S2** Differentially expressed genes after ozone treatment in Col‐0 and *defenseless*.

**Table S3** Differentially expressed genes between Col‐0 and *defenseless*.

**Table S4** Genes involved in ROS metabolism or scavenging.

**Table S5** List of 162 consistently downregulated genes in *defenseless* under control and ozone conditions; includes expression counts (used for Fig. 4 heatmap), gene descriptions, and GO terms.
Please note: Wiley is not responsible for the content or functionality of any Supporting Information supplied by the authors. Any queries (other than missing material) should be directed to the *New Phytologist* Central Office.

## Data Availability

The generated transcriptome RNA sequence data (raw data and the read‐count‐per‐gene data) have been deposited in the GEO database (NCBI) under accession GSE299572. Seeds of the mutants (*abi1‐1*, *defenseless*, and *eds1 sid2*) generated from this study have been deposited at the Arabidopsis Information Resource (https://www.arabidopsis.org) under the accession nos. (NASC codes: N2112652–N2112654; https://arabidopsis.info/StockInfo?NASC_id=2112653).
