# Supplementary material for: Decoding plant defense signaling using the defenseless mutant
Source: New Phytol. 2026 Jan 23;250(2):1141–56. doi: 10.1111/nph.70939 (PMC13001020; doi:10.1111/nph.70939)

***New Phytologist* Supporting Information**

Article title: Decoding plant defense signaling using the *defenseless* mutant

Authors: Bikash Baral and Mikael Brosché

Article acceptance date: 06 January 2026

The following Supporting Information is available for this article:

Methods S1 Experimental details and materials and methods for transcriptome analysis, abiotic and biotic stress assays.

Fig. S1 Growth and stress responses in *defenseless*. a) Fresh weight was measured in four-week-old plants from more than 77 plants. The fresh weight data was analyzed using a linear mixed-effects model with genotype as a fixed effect, and repeat group included as a random intercept to account for variation among experimental repeats. b) RT-qPCR was used to assess the relative expression of marker genes under control and ozone. Data represents five biological replicates, and statistical significance was determined using two-way ANOVA, followed by Tukey's test ($p < 0.05$). Samples with different letters are significantly different. c) Representative leaf images of Col-0 and mutants (*defenseless*, *cyp79b2b3*) infected with *Alternaria alternata*. The *cyp79b2b3* mutant showed the largest necrotic lesions, followed by *defenseless*, indicating increased susceptibility relative to Col-0. Plants aged 20–21 days were used. d) Whole-plant phenotypes after *A. alternata* or mock treatment. Mock-treated plants remained symptom-free, while infected plants displayed varying disease severity: *cyp79b2b3* > *defenseless* > Col-0. e) When Col-0 and *defenseless* mutants are transferred from clean growth rooms or chambers to the greenhouse, frequent appearance of unidentified pathogens was observed in *defenseless*.

Fig. S2 Flg22 induced ROS burst in Col-0, *defenseless* and *rbohD* from three biological repeats, each consisting of 24 leaf discs. Corresponding mock-treated controls are shown in small inset panels within each figure to illustrate basal ROS levels. The assay was performed in three independent biological experiments, and individual kinetic traces from each repeat are displayed to demonstrate experimental reproducibility. ROS production is expressed as relative light units (RLU) over time. Error bars represent SD.

Fig. S3 Leaf disc assay of Col-0 and *defenseless* under treatments with 3-AT (a) or SA (b). Data from six (3-AT) or eight (SA) biological repeats are presented as box-and-whisker plots ('*n*' = the number of leaf discs). Statistical significance was assessed using a one-way ANOVA, followed by Tukey's honestly

significant difference (HSD) post hoc test.

Fig. S4 Ascorbate redox status in Col-0 and *defenseless* under control conditions. Panel (a) shows reduced ascorbate (AsA_{Red}) levels, while panel (b) shows oxidized ascorbate (AsA_{Oxi}) measured in parallel extracts. Panel (c) depicts total ascorbate, representing the combined reduced and oxidized pools, and panel (d) displays dehydroascorbate (DHA) calculated from the total and reduced AsA values. Panel (e) illustrates the percentage of the total ascorbate pool present in the reduced form, whereas panel (f) shows the AsA_{Red}/AsA_{Oxi} redox ratio used to assess overall ascorbate redox status. Box plots show the distribution of biological replicates comparing Col-0 and *defenseless* genotypes under non-stress conditions. Statistical analysis was performed using a linear mixed-effects model with genotype treated as a fixed effect and repeat group as a random effect. Genotype effects were evaluated by ANOVA, followed by Tukey-adjusted pairwise comparisons of estimated marginal means. 'n = 80' represents the number of plants analyzed.

Fig. S5 Cuticle and NaCl responses in *defenseless*. a) Toluidine blue staining assessing cuticle permeability in Col-0, *defenseless*, and *abi1-1* mutants. Both mutants showed increased dye penetration, indicating compromised cuticle integrity, whereas Col-0 has limited staining, consistent with an intact cuticle. b, c) Effect of salt stress on root growth and viability. Seedlings of Col-0 and *defenseless* were grown on ½-strength MS medium supplemented with 0-150 mM NaCl. Primary root length was measured after 16 days (n = total plants across seven biological replicates). One-way ANOVA with Tukey's HSD test showed no significant differences in root length between genotypes at any salt concentration ($p > 0.05$). Concentrations above 125 mM NaCl were lethal, preventing germination or causing seedling death upon transfer. d) RT-qPCR was used to assess the relative expression of marker genes (*ERF109*, *RD20*, and *WRKY75*) under control and NaCl. Data represents seven biological replicates, and statistical significance was determined using two-way ANOVA, followed by Tukey's test ($p < 0.05$). Samples with different letters are significantly different.

Fig. S6 Transcriptomic profiles of selected genes induced under various treatment conditions, extracted from the Genevestigator Plants database.

Table S1-5 Supplementary tables are in large excel sheets, so they are submitted separately

Table S1 Primer sequences for genotyping and RT-qPCR, along with references.
Table S2 Differentially expressed genes after ozone treatment in Col-0 and defenseless.

Table S3 Differentially expressed genes between Col-0 and defenseless.

Table S4 Genes involved in ROS metabolism or scavenging.

Table S5 List of 162 consistently downregulated genes in defenseless under control and ozone conditions; includes expression counts (used for Fig. 4 heatmap), gene descriptions, and GO terms.

Methods S1 Experimental details and materials and methods for transcriptome analysis, abiotic and biotic stress assays.

Considerations for the crossing strategy

The original *coi1-16* also contains mutations in *gl1* and *pen2* (Ellis & Turner, 2002; Westphal *et al.*, 2008), these mutations were removed through backcross to Col-0. The *abi1-1* mutant was backcrossed three times to Col-0 before being used for further crosses and other experiments. We initially generated the mutants *coi1 ein2 sid2* (Xu *et al.*, 2015) and *coi1 eds1 ein2 sid2* (Overmyer *et al.*, 2018), which were used with crosses to *abi1-1* and *rbohD* to generate the sextuple *abi1-1 coi1 eds1 ein2 rbohD sid2*, which we will refer to as the *defenseless* mutant.

Inter-organelle redox communication between nucleus, chloroplasts, and mitochondria

The protocol for PAM (Pulse-Amplitude-Modulation) imaging and chemical treatments with methyl viologen (MV) and antimycin A (AA) has been previously published (Shapiguzov & Kangasjärvi, 2022). It was performed using an IMAGING-PAM M-Series (Walz, Germany). Leaf discs were punched from 26-28 day old plants and incubated overnight in darkness in MV (0.25 µM), AA (2.5 µM) or both in black 96-well plates. The MV stock is dissolved in water, while the AA stock is dissolved in DMSO. Thus, control leaf discs were incubated both in the presence and absence of DMSO (1:40000 v:v). In brief, the PAM protocol consists of cycles of blue light (450 nm, 80 µmol $m^{-2}\cdot s^{-1}$), followed by 20-min dark adaptation, and then measurement of Fv/Fm, see (Shapiguzov & Kangasjärvi, 2022) for more details. The protocol has 15 cycles, where the damage to photosystem II gets progressively worse for each cycle. The data was quantified from the fourth cycle from seven biological repeats, where each of the repeats had ten leaf discs for the treatments, six leaf discs for the water control, and 12 leaf discs for the DMSO control.

The same leaf disc protocol was also used for treatment with 5, 10, and 20 mM 3-AT (3-Amino-1,2,4-triazole) an inhibitor of catalase (Gechev *et al.*, 2002), and 0.1, 0.25 and 0.5 mM SA. Incubation with 3-AT was done in darkness overnight before start of the IMAGING-PAM protocol. For SA, after adding leaf discs to the 96-well plate, the plate was dark adapted for 20 minutes, followed by the IMAGING-PAM protocol. The 3-AT protocol was run at blue light levels 80 µmol $m^{-2}\cdot s^{-1}$ and the SA protocol at 130 µmol $m^{-2}\cdot s^{-1}$. Data was quantified from the 15th cycle.

RNA isolation and reverse transcription PCR (qRT-PCR)

For ozone treatments 22-d old plants were treated with 350 nl·L^{-1} ozone for 2 h. For NaCl treatments, a 10 µL drop of 150 mM NaCl or water, was placed on fully expanded leaves of 25-26-d old plants. Leaves were harvested after 6 h. Total RNA was extracted using the Spectrum Plant Total RNA Kit (Sigma-Aldrich) following the manufacturer's protocol. Three µg of RNA were treated with DNase I (Thermo Fisher Scientific) and reverse-transcribed using Maxima Reverse Transcriptase (Thermo Fisher Scientific). The resulting cDNA was diluted to a final volume of 100 µL, and 1 µL was used for qRT-PCR with 2× FastDye qPCR Master Mix (KleverLab) on a CFX Opus 384 System (Bio-Rad). Primer sequences and amplification efficiencies are detailed in Table S1. Relative expression levels were quantified using qBase+ v3.4 (CellCarta), with normalization to three reference genes (*PP2AA3*, *TIP41*, *YLS8*), whose stability (M-value) was assessed using geNorm. Statistical analysis was performed on $\log_{10}$-transformed data with five biological replicates (for ozone stress) or with seven biological replicates (for NaCl stress experiment) using two-way ANOVA, followed by Tukey's post hoc multiple comparisons test in Graphpad Prism 10.4.2.

RNA-seq and identification of differentially expressed genes

Plants were grown and treated with ozone as described above. Three biological repeats, with five plants per repeat, treated with O_3 for 2 h and corresponding controls were used for RNA-seq analysis. RNA was isolated with the Spectrum Plant Total RNA Kit (Sigma-Aldrich). RNA quality was assessed using the bioanalyzer RNA 6000 Nano assay (Agilent Technologies) to determine RNA integrity values (RIL). The library preparation and next-generation sequencing were done at Novogene GmbH, Germany. Sequence reads underwent pre-processing and quality assessment in adherence with established guidelines, employing FASTQC 0.12.0 and SortmeRNA v4.3.6 to remove RNA contaminants (Kopylova *et al.*, 2012). Subsequently, STAR-2.7.11b (Dobin *et al.*, 2013) was utilized for read mapping using the AtRTD3 transcriptome data (Zhang *et al.*, 2022)(https://ics.hutton.ac.uk/atRTD/RTD3/) as a reference, and the --quantMode GeneCounts to obtain the count number of reads per gene while mapping.

Counts were imported into R (v4.5.0; (R Core Team, 2024)) using Bioconductor v2.72.0 (Gentleman *et al.*, 2004). For quality assessment and visualization, read counts were normalized to variance-stabilized counts (VST) using the Bioconductor DESeq2 package v1.12.0 (Love *et al.*, 2014). Biological relevance, including replicate similarity, was assessed using multi-dimensional scaling (MDS) with Euclidean distance, and visualized through heatmaps (pheatmap), Venn diagrams (VennDiagram), and gene ontology analysis (clusterProfiler::enrichGO()), with data wrangling via dplyr and plotting using ggplot2. Samples with a correlation coefficient below 0.8 were excluded from further analysis. Differentially expressed genes (DEGs) were selected with a $\log_2$ fold-change threshold of 1 and an adjusted *p*-value cut-

off of 0.05. Low VST expression values (average biological replicates ≤1 across all sampling times) were filtered out. See Tables S2 and S3 for a list of all genes.

Gene ontology (GO) enrichment analysis within the Biological Process (BP) category was performed using the clusterProfiler package in RStudio. DEGs were identified using the DESeq2 package, and the GO enrichment results were visualized using the ggplot2 package.

Infections with *Alternaria*

Three-week-old Col-0, *defenseless*, and *cyp79b2b3* plants were grown as described above and then moved to a secondary growth chamber (Fitotron® SGC120, Weiss Technik; 12 h light/dark cycle) before infection. Mature spores of *Alternaria alternata*, cultured on homemade potato dextrose agar (PDA), were used. The PDA consisted of 300 g grated potato, 50 g grated tomato, and 25 g grated carrot boiled in 750 mL MQ water, filtered, adjusted to 1 L, and autoclaved with 15 g agar. Agar plates were inoculated with 8 mm pieces of *Alternaria* and incubated in the dark for one month for them to sporulate. Spores of *Altenaria* were harvested with sterile scalpels, filtered through Miracloth to remove mycelium, scored with a hemocytometer, and diluted to 1×10^6 spores/mL. Potato dextrose broth (PDB) purchased from Sigma-Aldrich® Solutions, Germany, served as the control or Mock treatment.

Altenaria alternata infections were performed within a sterile laminar flow hood. Ten plants, each with four fully expanded leaves (totaling 40 leaves), were inoculated on the adaxial leaf surface with 5 µL of spore suspension (1×10^6 spores/mL). Mock inoculations were performed using PDB as a control. Post-inoculation, plants were enclosed in mini greenhouses with adequate water to maintain high humidity and prevent cross-contamination. Plants were monitored in the growth chamber for the progression of the fungal lesion symptoms. Seven days post-infection, infected leaves were excised, photographed (Nikon D3100), and lesion areas were quantified as a percentage of the total plant area using LifeSize software (https://vittorioaccomazzi.github.io/LeafSize/).

Infections with *Pseudomonas*

Overnight cultures of *Pseudomonas syringae* pv. Tomato DC3000 was grown at 28°C in liquid LB, and subsequently diluted 1:50 in fresh LB medium, followed by incubation for an additional 12 h. Bacterial cells were harvested by centrifugation (1,500 × *g*, 2 min), resuspended, and washed twice with 10 mM $MgCl_2$. The bacterial density was adjusted to $OD_{600} = 0.2$ (corresponding to ~1×10^8 CFU/mL) and prepared for spray inoculation in 10 mM $MgCl_2$ containing 0.02% (v/v) Silwet L-77. Spray inoculation was performed using an airbrush, with infections performed at midday. As a mock control, the same

amount of the buffer with Silwet was used. To maintain optimal humidity, plant trays were covered for 2 h before and after inoculation. Plants were cultivated in a Fitotron® SGC120 (Weiss Technik) under a 12 h light (170 µmol $m^{-2} \cdot s^{-1}$)/12 h dark cycle at 23°C/19°C (day/night) and 60%/70% relative humidity. All experiments used 3.5-week-old plants.

For bacterial quantification, three leaves per plant were surface-sterilized with 70% ethanol, rinsed in sterile Milli-Q water, and sampled using 0.7 cm cork borers. Leaf discs were homogenized in 800 µL of 10 mM $MgCl_2$, serially diluted (10^{-4} to 10^{-7}), and 10 µL of the dilution was plated on Lysogeny broth agar (LB-agar) media. Leaf sampling was performed at 24 h, 48 h, 72 h, and 96 h post-infection. Each replicate consisted of eight biological replicates, and the entire experiment was repeated three times (3 repeat groups; 8 samples each). Bacterial colonies were allowed to grow for 40-48 h at 28°C until clearly visible and were manually quantified. When necessary, a stereoscope (Zeiss Stemi 2000-C Trinocular Microscope, UK) was used to score the bacterial colonies.

Toluidine blue staining

To assess cuticle permeability, toluidine blue (TB) staining was performed following the protocol of Tanaka *et al*., (2004) and Bessire *et al*., (2007). Fully expanded leaves of 3-week-old plants were treated with 0.05% TB solution (10 µL) on the adaxial surface, excluding the midrib, and incubated under a transparent cover for 2 h. After rinsing with water, stained areas were photographed using a camera (Nikon D3100, Japan) and quantified using ImageJ software.

Quantification of ozone damage

22-23 days old Col-0, *abi1-1*, and *defenseless* were treated with a 6 h ozone, 350 $nL \cdot L^{-1}$. Post-treatment, rosettes were harvested in 15 mL of Milli-Q water in a 50 mL Falcon tube. Conductivity ($mS \cdot cm^{-1}$) was measured using a conductivity meter (Mettler Toledo) following brief vortexing. Total ion content was determined after samples were frozen at -20°C overnight, thawed, and vortexed again under the same conditions. Ion leakage was calculated as Ion leakage (%) = (Initial conductivity / Total conductivity) × 100.

Quantification of waterloss

25-26 days old Col-0, *abi1-1*, and *defenseless* mutants were cultivated under previously described growth conditions and subsequently subjected to water-loss assays. For each experiment, two-three fully expanded leaves were excised per plant, and their fresh weight was immediately recorded post-excision. The excised leaves were allowed to transpire (with the abaxial side of the leaf facing up) under ambient

laboratory conditions for 2 h. Following this period, the final leaf weight was measured. Water loss was quantified as the percentage reduction in initial fresh weight.

Salt root growth

Col-0 and *defenseless* were exposed to a gradient of sodium chloride (NaCl) concentrations: 0 mM (control), 25 mM, 50 mM, 75 mM, 100 mM, 125 mM, and 150 mM. Seedlings were initially germinated on ½-strength MS medium for 7 d under controlled conditions (as mentioned previously). Following initial growth, uniform seedlings were transferred to ½-strength MS agar plates supplemented with the respective NaCl concentrations (from 25-150 mM NaCl). Multiple biological replicates (7 repeat groups) were prepared for each treatment. Plates were maintained in a growth chamber under identical environmental conditions for an additional 14-d. During this period, plant development was monitored, and phenotypic observations were documented. At the end of the treatment period, to determine primary root lengths, plates were photographed, and root systems were imaged and quantitatively assessed using ImageJ software.

Fresh weight measurement

Growth phenotypes of Col-0, *defenseless*, and *abi1-1* were assessed by measuring the fresh weight of rosettes from 25-28 d-old plants at the same developmental stage. Whole rosettes were excised, debris was gently cleaned and weighed immediately. Fresh-weight data were collected from four independent biological experiments comprising over 77 plants.

ROS assay

ROS production in Arabidopsis leaf discs was quantified using a luminol-horseradish peroxidase (HRP) chemiluminescence assay following the standard protocols (Albert *et al.*, 2015; Bisceglia *et al.*, 2015). Leaf discs of 6 mm in diameter from the fully expanded leaves were excised from 28-day-old plants one day before the measurement, washed, and incubated for 2 h in sterile water before transferring single discs (adaxial side up) into 96-well plates containing 200 µL water. Plates were kept under continuous light for 16-20 h to minimize wound-induced ROS.

Immediately before measurement, water was removed, and 200 µL of an elicitation solution containing luminol (64 µM), Horseradish Peroxidase (HRP, 0.15 µM), and flg22 (0.66 µM) at the mentioned final concentration was added. For the control, MiliQ water of the same volume was added. Luminescence was recorded using an EnSpire plate reader (Perkin Elmer), with 1 sec integration time after photomultiplier tube (PMT) activation. ROS kinetics were measured over time and expressed as relative light units (RLU), reflecting luminol oxidation in the presence of apoplastic hydrogen peroxide.

Ascorbate redox profiling

Ascorbate redox profiling was performed using the microplate-based colorimetric assay devised by Gillespie *et al*., (2007). Ascorbate concentrations were calculated from standard curves, and dehydroascorbate was determined as total ascorbate minus reduced ascorbate.

References

Albert M, Butenko M, Aalen R, Felix G, Wildhagen M. 2015. Chemiluminescence Detection of the Oxidative Burst in Plant Leaf Pieces. *BIO-PROTOCOL* 5.

Bessire M, Chassot C, Jacquat AC, Humphry M, Borel S, Petétot JMDC, Métraux JP, Nawrath C. 2007. A permeable cuticle in Arabidopsis leads to a strong resistance to Botrytis cinerea. *EMBO Journal* 26: 2158–2168.

Bisceglia N, Gravino M, Savatin D. 2015. Luminol-based Assay for Detection of Immunity Elicitor-induced Hydrogen Peroxide Production in Arabidopsis thaliana Leaves. *BIO-PROTOCOL* 5.

Dobin A, Davis CA, Schlesinger F, Drenkow J, Zaleski C, Jha S, Batut P, Chaisson M, Gingeras TR. 2013. STAR: ultrafast universal RNA-seq aligner. *Bioinformatics (Oxford, England)* 29: 15–21.

Ellis C, Turner JG. 2002. A conditionally fertile coi1 allele indicates cross-talk between plant hormone signalling pathways in Arabidopsis thaliana seeds and young seedlings. *Planta* 215: 549–56.

Gechev T, Gadjev I, Van Breusegem F, Inzé D, Dukiandjiev S, Toneva V, Minkov I. 2002. Hydrogen peroxide protects tobacco from oxidative stress by inducing a set of antioxidant enzymes. *Cellular and molecular life sciences: CMLS* 59: 708–14.

Gentleman RC, Carey VJ, Bates DM, Bolstad B, Dettling M, Dudoit S, Ellis B, Gautier L, Ge Y, Gentry J, *et al.* 2004. Bioconductor: open software development for computational biology and bioinformatics. *Genome biology* 5: R80.

Gillespie KM, Ainsworth EA. 2007. Measurement of reduced, oxidized and total ascorbate content in plants. *Nature Protocols* 2: 871–874.

Kopylova E, Noé L, Touzet H. 2012. SortMeRNA: fast and accurate filtering of ribosomal RNAs in metatranscriptomic data. *Bioinformatics* 28: 3211–3217.

Love MI, Huber W, Anders S. 2014. Moderated estimation of fold change and dispersion for RNA-seq data with DESeq2. *Genome Biology* 15: 550.

Overmyer K, Vuorinen K, Brosché M. 2018. Interaction points in plant stress signaling pathways. *Physiologia plantarum* 162: 191–204.

R Core Team. 2024. R: A language and environment for statistical computing. R foundation for statistical computing.

Shapiguzov A, Kangasjärvi J. 2022. Studying plant stress reactions in vivo by PAM chlorophyll fluorescence imaging. *Methods in molecular biology (Clifton, N.J.)* 2526: 43–61.

Tanaka T, Tanaka H, Machida C, Watanabe M, Machida Y. 2004. A new method for rapid visualization of defects in leaf cuticle reveals five intrinsic patterns of surface defects in *Arabidopsis*. *The Plant Journal* 37: 139–146.

Westphal L, Scheel D, Rosahl S. 2008. The *coi1-16* Mutant Harbors a Second Site Mutation Rendering PEN2 Nonfunctional. *The Plant Cell* 20: 824–826.

Xu E, Vaahtera L, Brosché M. 2015. Roles of Defense Hormones in the Regulation of Ozone-Induced Changes in Gene Expression and Cell Death. *Molecular Plant* 8: 1776–1794.

Zhang R, Kuo R, Coulter M, Calixto CPG, Entizne JC, Guo W, Marquez Y, Milne L, Riegler S, Matsui A, *et al.* 2022. A high-resolution single-molecule sequencing-based Arabidopsis transcriptome using novel methods of Iso-seq analysis. *Genome Biology* 23: 149.

Fig. S1 Growth and stress responses in *defenseless*. a) Fresh weight was measured in four-week-old plants from more than 77 plants. The fresh weight data was analyzed using a linear mixed-effects model with genotype as a fixed effect, and repeat group included as a random intercept to account for variation among experimental repeats. b) RT-qPCR was used to assess the relative expression of marker genes under control and ozone. Data represents five biological replicates, and statistical significance was determined using two-way ANOVA, followed by Tukey's test ($p < 0.05$). Samples with different letters are significantly different. c) Representative leaf images of Col-0 and mutants (*defenseless*, *cyp79b2b3*) infected with *Alternaria alternata*. The *cyp79b2b3* mutant showed the largest necrotic lesions, followed by *defenseless*, indicating increased susceptibility relative to Col-0. Plants aged 20-21 days were used. d) Whole-plant phenotypes after *A. alternata* or mock treatment. Mock-treated plants remained symptom-free, while infected plants displayed varying disease severity: *cyp79b2b3* > *defenseless* > Col-0. e) When Col-0 and *defenseless* mutants are transferred from clean growth rooms or chambers to the greenhouse, frequent appearance of unidentified pathogens was observed in *defenseless*.

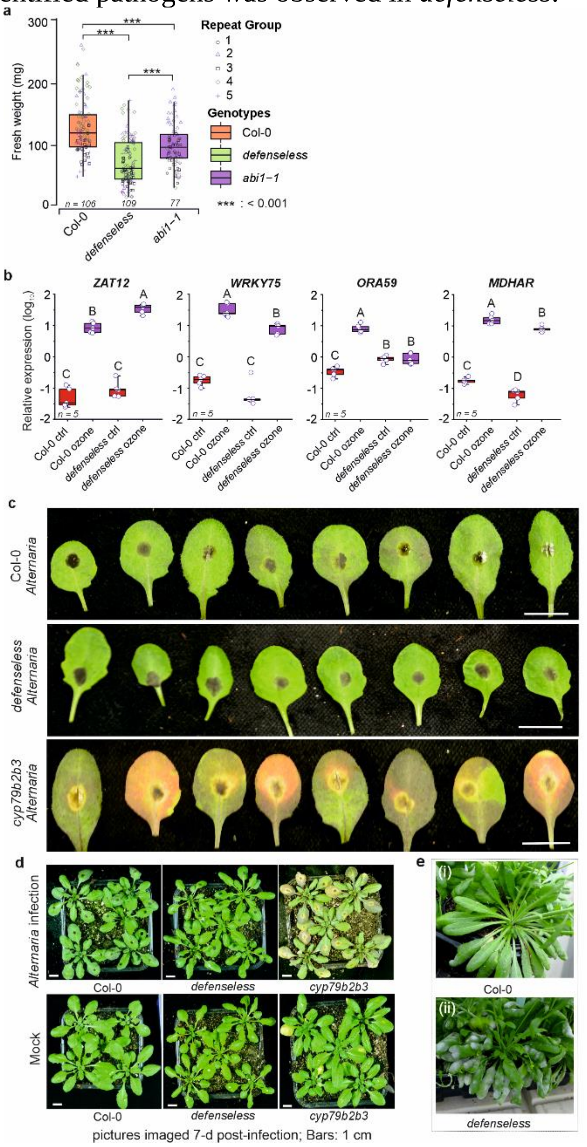

Fig. S2 Flg22 induced ROS burst in Col-0, *defenseless* and *rbohD* from three biological repeats, each consisting of 24 leaf discs. Corresponding mock-treated controls are shown in small inset panels within each figure to illustrate basal ROS levels. The assay was performed in three independent biological experiments, and individual kinetic traces from each repeat are displayed to demonstrate experimental reproducibility. ROS production is expressed as relative light units (RLU) over time. Error bars represent SD.

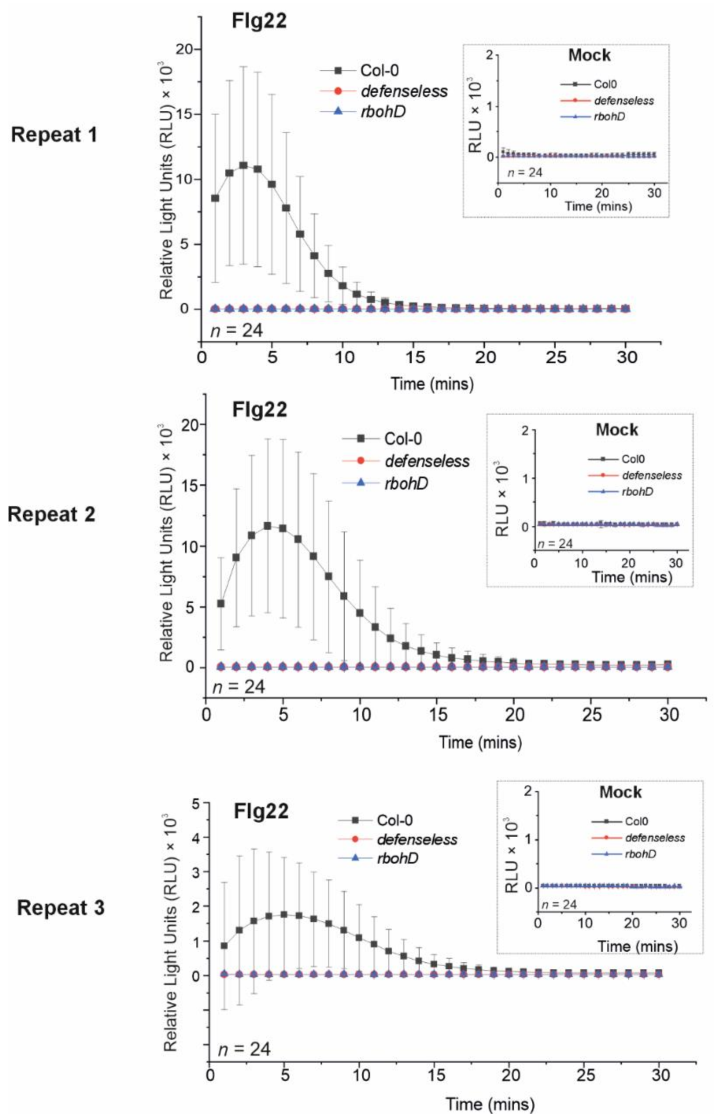

Fig. S3 Leaf disc assay of Col-0 and *defenseless* under treatments with 3-AT (a) or SA (b). Data from six (3-AT) or eight (SA) biological repeats are presented as box-and-whisker plots ('*n*' = the number of leaf discs). Statistical significance was assessed using a one-way ANOVA, followed by Tukey's honestly significant difference (HSD) post-hoc test.

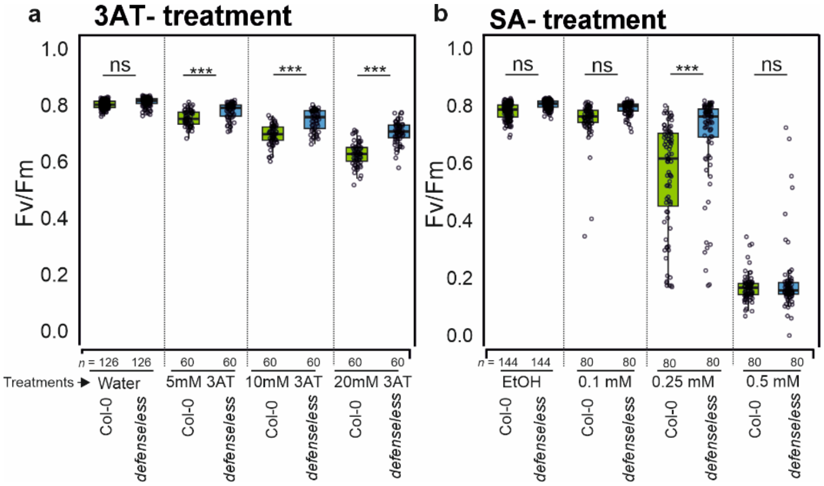

Fig. S4 Ascorbate redox status in Col-0 and *defenseless* under control conditions. Panel (a) shows reduced ascorbate (AsA_{Red}) levels, while panel (b) shows oxidized ascorbate (AsA_{Oxi}) measured in parallel extracts. Panel (c) depicts total ascorbate, representing the combined reduced and oxidized pools, and panel (d) displays dehydroascorbate (DHA) calculated from the total and reduced AsA values. Panel (e) illustrates the percentage of the total ascorbate pool present in the reduced form, whereas panel (f) shows the AsA_{Red}/AsA_{Oxi} redox ratio used to assess overall ascorbate redox status. Box plots show the distribution of biological replicates comparing Col-0 and *defenseless* genotypes under non-stress conditions. Statistical analysis was performed using a linear mixed-effects model with genotype treated as a fixed effect and repeat group as a random effect. Genotype effects were evaluated by ANOVA, followed by Tukey-adjusted pairwise comparisons of estimated marginal means. 'n = 80' represents the number of plants analyzed.

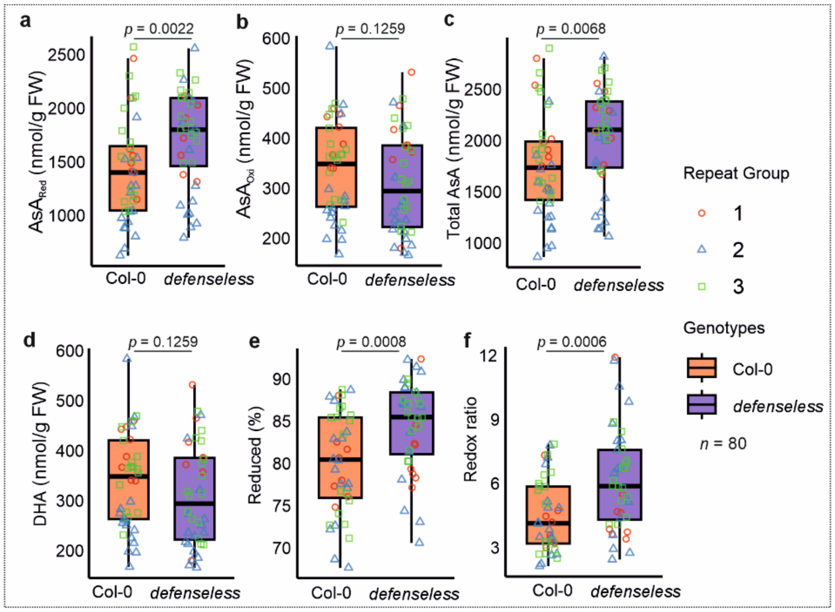

Fig. S5 Cuticle and NaCl responses in *defenseless*. a) Toluidine blue staining assessing cuticle permeability in Col-0, *defenseless*, and *abi1-1* mutants. Both mutants showed increased dye penetration, indicating compromised cuticle integrity, whereas Col-0 has limited staining, consistent with an intact cuticle. b, c) Effect of salt stress on root growth and viability. Seedlings of Col-0 and *defenseless* were grown on ½-strength MS medium supplemented with 0-150 mM NaCl. Primary root length was measured after 16 days (n = total plants across seven biological replicates). One-way ANOVA with Tukey's HSD test showed no significant differences in root length between genotypes at any salt concentration ($p > 0.05$). Concentrations above 125 mM NaCl were lethal, preventing germination or causing seedling death upon transfer. d) RT-qPCR was used to assess the relative expression of marker genes (*ERF109*, *RD20*, and *WRKY75*) under control and NaCl. Data represents seven biological replicates, and statistical significance was determined using two-way ANOVA, followed by Tukey's test ($p < 0.05$). Samples with different letters are significantly different.

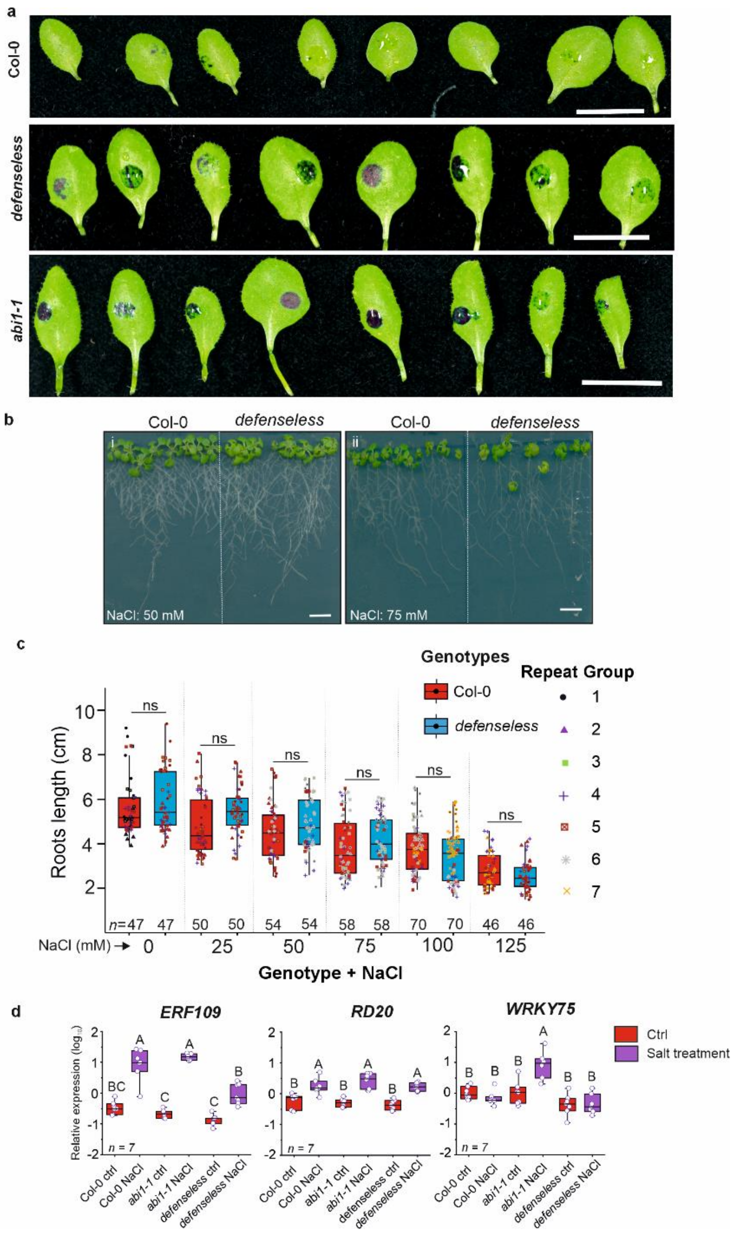

Fig. S6 Transcriptomic profiles of selected genes induced under various treatment conditions, extracted from the Genevestigator Plants database.

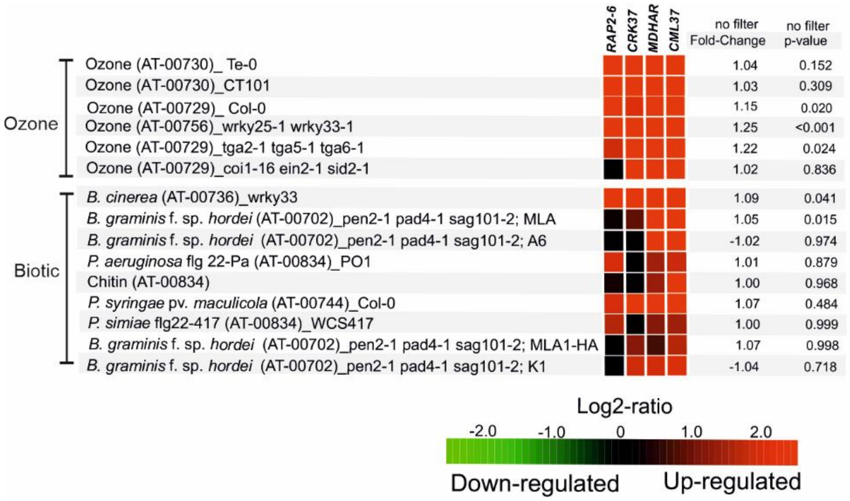

Supplement: Supplementary file 1 — Fig. S1 Growth and stress responses in defenseless. Fig. S2 Flg22 induced ROS burst in Col‐0, defenseless and rbohD from three biological repeats, each consisting of 24 leaf disks. Fig. S3 Leaf disk assay of Col‐0 and defenseless under treatments with 3‐AT (a) or SA (b). Fig. S4 Ascorbate redox status in Col‐0 and defenseless under control conditions. Fig. S5 Cuticle and NaCl responses in defenseless. Fig. S6 Transcriptomic profiles of selected genes induced under various treatment conditions, extracted from the Genevestigator Plants database. Methods S1 Experimental details and materials and methods for transcriptome analysis, abiotic and biotic stress assays. [file NPH-250-1141-s006.pdf]
